# Repositioning of a novel GABA-B receptor agonist, AZD3355 (Lesogaberan), for the treatment of non-alcoholic steatohepatitis

**DOI:** 10.1038/s41598-021-99008-2

**Published:** 2021-10-21

**Authors:** Dipankar Bhattacharya, Christine Becker, Benjamin Readhead, Nicolas Goossens, Jacqueline Novik, Maria Isabel Fiel, Leslie P. Cousens, Björn Magnusson, Anna Backmark, Ryan Hicks, Joel T. Dudley, Scott L. Friedman

**Affiliations:** 1 Division of Liver Diseases, Icahn School of Medicine at Mount Sinai, Box 1123, 1425 Madison Ave. Room 1170, New York, NY 10029 USA; 2 Department of Genetics and Genomic Sciences, Icahn School of Medicine at Mount Sinai, New York, NY USA; 3 Division of Clinical Immunology, Icahn School of Medicine at Mount Sinai, New York, NY USA; 4 Department of Pathology, Icahn School of Medicine at Mount Sinai, New York, NY USA; 5 Emerging Innovations, Discovery Sciences, R&D, AstraZeneca, Boston, MA USA; 6 Discovery Biology, Discovery Sciences, R&D, AstraZeneca, Gothenburg, Sweden; 7 BioPharmaceuticals R&D Cell Therapy, Research and Early Development, Cardiovascular, Renal and Metabolism (CVRM), BioPharmaceuticals R&D, AstraZeneca, Gothenburg, Sweden; 8 Present Address: Arizona State University-Banner Neurodegenerative Disease Research Center, Arizona, USA; 9 Present Address: Division of Gastroenterology, Geneva University Hospital, Geneva, Switzerland

**Keywords:** Metabolic disorders, Drug discovery, Gastroenterology

## Abstract

Non-alcoholic steatohepatitis (NASH) is a rising health challenge, with no approved drugs. We used a computational drug repositioning strategy to uncover a novel therapy for NASH, identifying a GABA-B receptor agonist, AZD3355 (Lesogaberan) previously evaluated as a therapy for esophageal reflux. AZD3355’s potential efficacy in NASH was tested in human stellate cells, human precision cut liver slices (hPCLS), and in vivo in a well-validated murine model of NASH. In human stellate cells AZD3355 significantly downregulated profibrotic gene and protein expression. Transcriptomic analysis of these responses identified key regulatory nodes impacted by AZD3355, including Myc, as well as MAP and ERK kinases. In PCLS, AZD3355 down-regulated collagen1α1, αSMA and TNF-α mRNAs as well as secreted collagen1α1. In vivo, the drug significantly improved histology, profibrogenic gene expression, and tumor development, which was comparable to activity of obeticholic acid in a robust mouse model of NASH, but awaits further testing to determine its relative efficacy in patients. These data identify a well-tolerated clinical stage asset as a novel candidate therapy for human NASH through its hepatoprotective, anti-inflammatory and antifibrotic mechanisms of action. The approach validates computational methods to identify novel therapies in NASH in uncovering new pathways of disease development that can be rapidly translated into clinical trials.

## Introduction

Nonalcoholic steatohepatitis (NASH) is a serious and escalating health threat in the United States, affecting between 6.5 million to 16.3 million Americans^1^, with a rising incidence paralleling the obesity epidemic. NASH represents a more advanced form of non-alcoholic fatty liver disease (NAFLD), which affects up to 60 million Americans and hundreds of millions worldwide^2,3^. NASH disease is characterized by hepatocellular fat, inflammation and injury with accumulation of fibrillar collagen generated by activated hepatic stellate cells, the principle fibrogenic cell in liver^4^. NASH can lead to liver failure and hepatocellular carcinoma (HCC)^5^, and will supplant hepatitis C as the primary indication for liver transplantation in the coming years, along with alcoholic liver disease, which has many overlapping pathogenic pathways with NASH^6^.

The expanding public health impact of NASH underscores the urgent need to develop novel therapies that prevent progression and/or reverse fibrosis to improve outcomes; however, there are no approved therapies to date. An incomplete understanding of molecular mechanisms underlying NASH is a major impediment to therapeutic development. Further, a number of immune, metabolic, fibrotic, and other major pathways contribute to NASH pathogenesis^4^ adding to the complexity of identifying target-based therapeutic mechanisms. Obeticholic acid (OCA or INT-747 or 6alpha-ethylchenodeoxycholic acid (6-ECDCA)) is the most advanced investigational drug for NASH thus far, but is not yet FDA-approved due to safety concerns. Moreover, this agent improves NASH fibrosis in only 23.1% of patients in a phase 3 trial, compared to 11.9% of placebo-treated patients^7^.

In drug development only 6–7% of drugs that successfully enter Phase I trials advance to FDA approval^8,9^. The major reasons for drug failures include adverse effects (toxicity), inadequate efficacy, off-target effects and poor pharmacokinetic (DMPK) profiling^10–12^.

More importantly, many drugs that have clean safety profiles are shelved because of insufficient efficacy or commercial decisions related to the available market and competitive landscape. Some of these agents can be repurposed for treating diseases unrelated to their original clinical indication based on an emerging understanding of pathobiology, and enhanced by computational tools to search vast databases of publicly available knowledge^13,14^. These approaches identify connections between drug action and disease that may not be intuitive to even the most knowledgeable physician-scientist. Repurposing can have major advantages in time- and cost-efficiency. For example, Phase 1 safety studies of the original clinical development program can be utilized to assess the risk–benefit ratio a newly proposed indication, and where deemed appropriate, accelerate into Phase 2 testing of efficacy to significantly reduce development timelines and costs^15^. In addition, drug repurposing reduces risk of failure from toxicity and can lower the cost of drug development since these FDA-approved drugs have passed regulatory scrutiny and sometimes post-market surveillance as well.

Traditional drug discovery relies on target-based or phenotypic screening, with further development largely driven by clinical observations and pharmacology. Recent evidence suggests that powerful bioinformatic methods applied to systematically mine the wealth of available genomic, molecular, and clinical data can identify additional connections between disease and drug signatures that are not mechanistically intuitive, but that implicate novel indications and/or disease subsets. The potential to develop repurposed drugs to treat NASH has been buoyed by the development of analytic methods to mine transcriptomic data for evidence of a therapeutic signal^16^. Mining large volumes of genomic, molecular, and clinical data offers new approaches for the generation of novel, testable hypotheses connecting drug activity with unmet clinical needs. High-dimensional molecular data, such as transcriptomic profiling, are routinely used to generate rich perspectives of biological states of interest and offer exciting opportunities to exploit computational methods to better understand human response to disease and treatment. A powerful example of this is computational drug repositioning, which leverages systematic approaches to characterize and relate disease and drug-induced states to identify novel indications for existing therapies, thus prioritizing valuable experimental validation efforts. The aim of computational drug repurposing is the more rapid and systematic identification of novel therapeutic strategies compared to traditional modes of drug development.

In the current study we used a computational drug repurposing strategy to identify an FDA-approved gamma-aminobutyric acid receptor B (GABA-B) agonist as a potential new therapy for NASH. We then explored the potential efficacy of AZD3355 (Lesogaberan)^17,18^, an agent that previously failed in a Phase 2b human trial for treatment of gastroesophageal reflux disease (GERD)^19^, as a novel treatment for NASH using a combination of culture and in vivo studies.

## Results

### Computational drug repurposing identifies AZD3355 as a potential treatment for NASH

**Figure 1:**
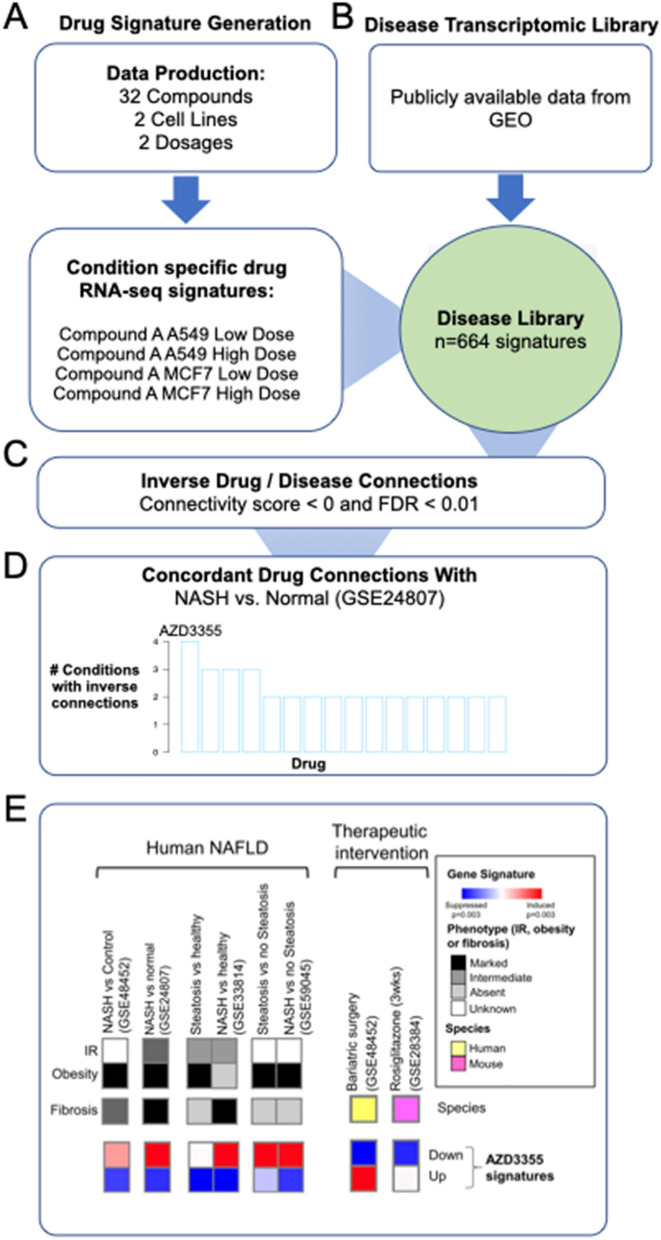
Computational drug repurposing identifying AZD3355 as a potential treatment for NASH. (**A**) We generated RNA-sequence profiles for 32 drugs, across four conditions to define condition specific drug signatures, (**B**) which were then compared with a large library of publicly available disease gene expression profiles using a connectivity mapping approach. (**C**,**D**) Significant (FDR < 0.01), inverse connections were found between all four AZD3355 signatures and a NASH gene expression signature (GSE24807). (**E**) In silico validation of AZD3355 in additional human and therapeutic NAFLD hepatic transcriptomic datasets. AZD3355 perturbation signatures were inversely enriched in human NAFLD (left), and positively enriched in datasets testing previously validated NAFLD therapies in human and mice (right). Abbreviations: IR, insulin resistance.

Using a connectivity mapping approach, we generated RNA-sequence profiles on a defined set of 32 clinic-ready compounds, under 4 conditions (e.g. “Compound-A A549 High dose”, “Compound-A MCF7 High dose”, “Compound-A A549 Low dose”, “Compound-A MCF7 Low dose”), and compared these to cell-matched DMSO-treated control samples to define a differential gene expression signature for each (Fig. 1A). Each compound-condition was then compared to a disease gene expression library (generated from publicly available data^20,21^) and a connectivity score was obtained for each compound-condition/disease pair (Fig. 1B).

Given the high unmet need to develop novel therapeutic strategies for improved treatment of NASH, we were interested to note significant inverse connections (FDR < 0.01) between all four AZD3355 signatures and a NASH gene expression signature (GSE24807). Of the 32 compounds profiled within our study, only AZD3355 demonstrated this concordant inverse connectivity across all four experimental conditions (Fig. 1C–D).

To further evaluate whether the transcriptomic relationship between AZD3355 and the NASH gene expression signature was unusually strong, we performed the same repurposing analysis for all 1,309 compounds (including many FDA approved compounds) in Connectivity Map (a collection of genome-wide transcriptional expression data from cultured human cells treated with bioactive small molecules) against the NASH signature and found that the connectivity score for AZD3355 was in the top 2% of predictions for NASH when ranked among all 1,309 compounds. Therefore, we regarded the connection between AZD3355 and NASH to be significant, even in the context of a large set of compounds with diverse mechanisms and disease indications.

To further investigate the context of AZD3355’s effects upon NASH biology, we tested the AZD3355 gene signatures against additional human NAFLD liver and NAFLD therapeutic transcriptomic datasets (Fig. 1E). We observed consistent inverse expression of gene signatures between AZD3355 and human NASH. Furthermore, we found enrichment of the AZD3355 gene signatures in hepatic transcriptomic datasets of therapeutic interventions known to improve NAFLD (bariatric surgery and rosiglitazone therapy). These findings suggest that AZD3355 induces a gene signature similar to candidate therapies of NASH and opposite to human NASH, thus establishing the rationale to directly test AZD3355’s activity in models of fibrosis and NASH in culture and in vivo.

### AZD3355 attenuates hepatic stellate cell activation

**Figure 2:**
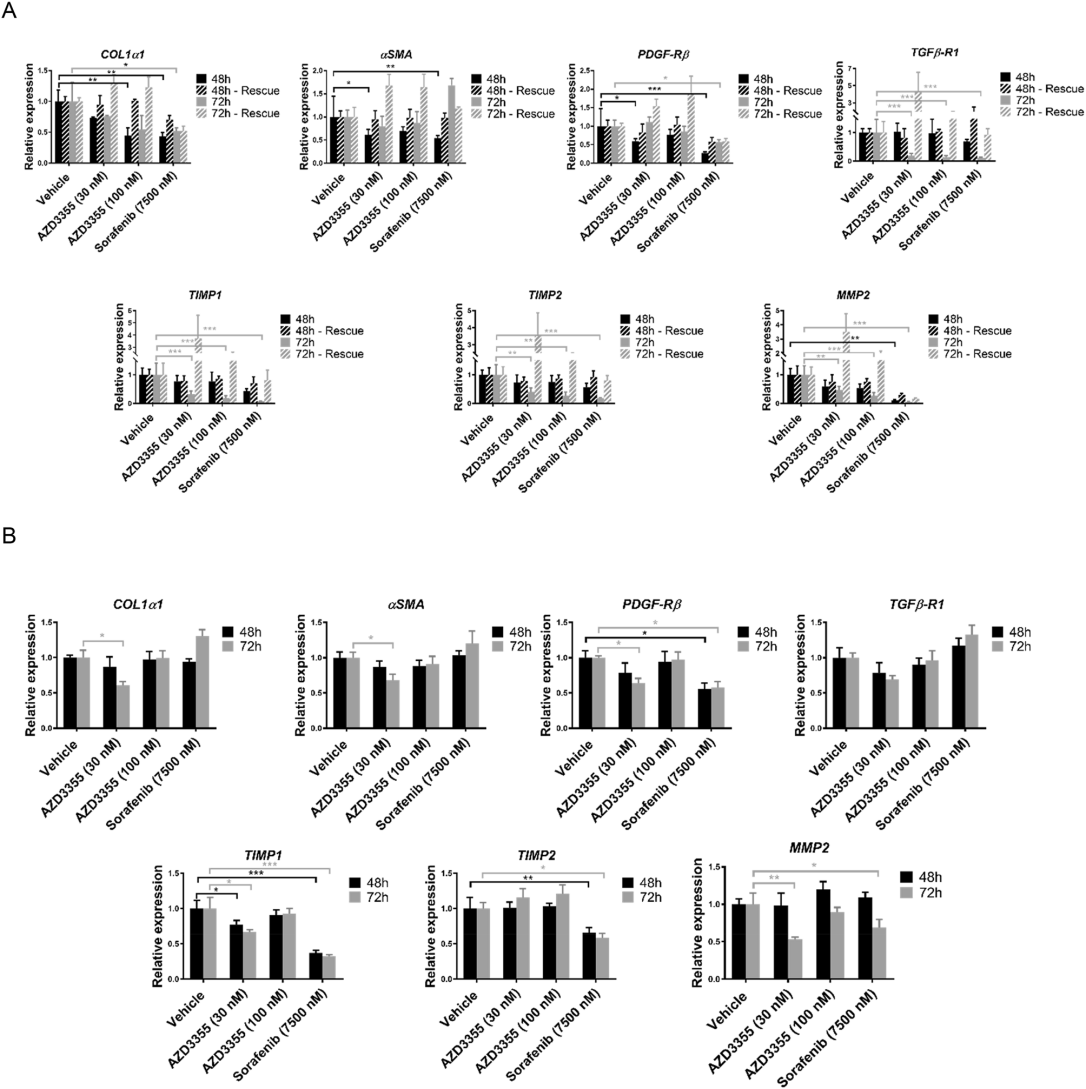
AZD3355 down-regulates activation-related mRNAs in HSCs. LX-2 cells (**A**) were treated with either DMSO (as vehicle) or either AZD3355 (30 nM or 100 nM) and 7500 nM Sorafenib (as positive control) for 48 and 72 h. The vehicle or drugs were withdrawn after 48 and 72 h and cells were maintained in DMEM media with 10% FBS for an additional 48 and 72 h, respectively. Dose-dependent down-regulation of fibrogenic related mRNA expression was observed in response to AZD3355 treatment. Recovery of gene expression in (striped bars) after drug removal (solid bars) indicates that down-regulation was reversible and not due to toxicity. Primary human hepatic stellate cells (**B**) were treated as described for as LX-2 cells (without rescue). Stellate cell activation genes were significantly down-regulated in 30 nM AZD3355 at 72 h treatment. Results are reported as means ± SEM (n = 3). *p < 0.05, **p < 0.01, ***p < 0.001.

We assessed the effects of AZD3355 on hepatic stellate cell gene expression in both LX-2 cells, an immortalized human stellate cell line^22^ and primary human hepatic stellate cells (phHSCs) to assess the drug's impact on fibrogenesis. Cells were treated with 30 nM and 100 nM AZD3355 for 48 and 72 h. Both LX-2 and phHSCs were culture-activated through growth on uncoated plastic in the presence of serum, and expressed high level of key profibrotic genes in the absence of additional stimuli. DMSO and the multi-kinase inhibitor sorafenib (7,500 nM) were used as vehicle and as a positive control, respectively. In LX-2 cells, there was down-regulation of profibrotic genes by AZD3355 (Fig. 2A, solid bar). To exclude toxicity as a reason for reduced gene expression, fresh media without drug was restored to the cells after 48 and 72 h, respectively, and 72 h after that mRNA expression resumed baseline levels (Fig. 2A, striped bar).

In parallel, we evaluated the effects of AZD3355 on phHSCs. The cells were incubated with 30 nM or 100 nM AZD3355 48 and 72 h, compared to vehicle. Gene expression for *COL1α1*, *αSMA*, *β-PDGFR*, *TGFβ-R1*, *TIMP1* and *MMP2* was significantly down-regulated by 30 nM (Fig. 2B). Selection of optimal effective concentrations of AZD3355 was determined by cytotoxicity, proliferation and apoptosis assays; both LX-2 and phHSCs tolerated up to 100 nM AZD3355 for 72 h (Supplemental Figure S2A and B).

### Transcriptomic / pathway analysis in phHSCs in response to AZD3355

**Figure 3:**
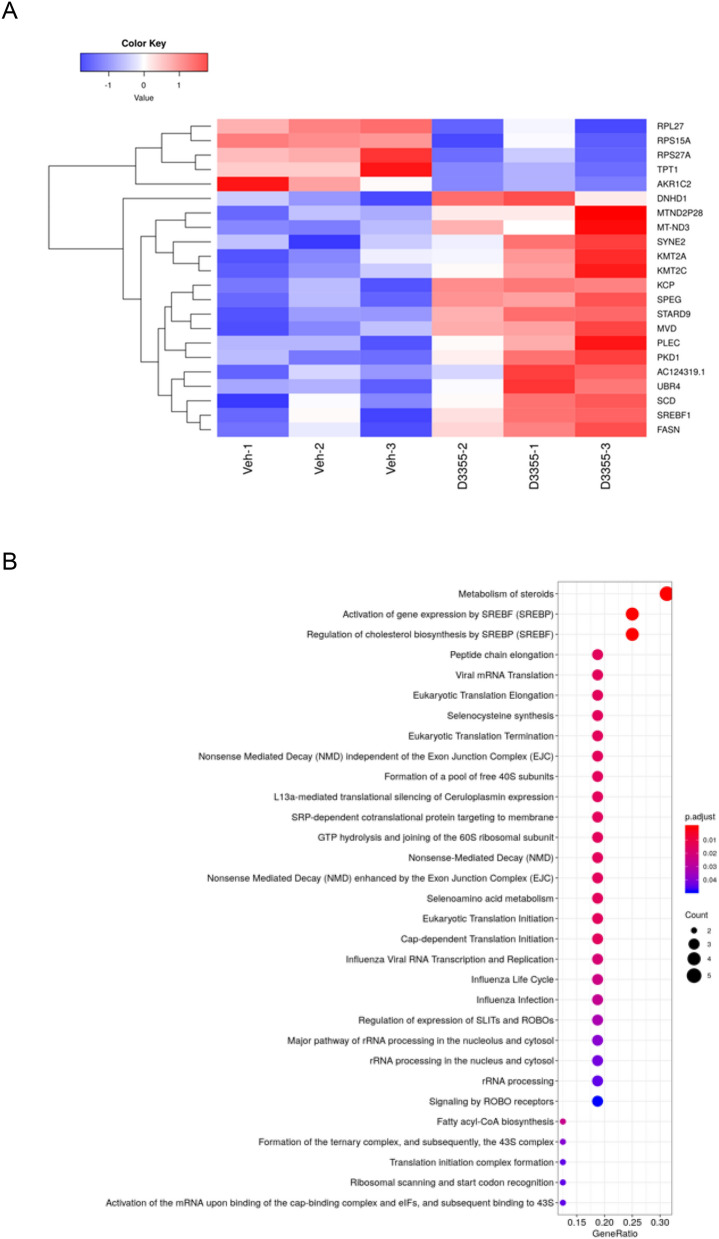
Differential gene expression and molecular pathway analysis in primary human hepatic stellate cells treated with AZD3355. Primary human hepatic stellate cells were treated with either DMSO (as vehicle) or AZD3355 (30 nM) for 72 h. A heatmap was generated from RNA-seq data (**A**) summarizing -significantly differentially expressed genes (padj < 0.1) in AZD3355 treated cells compared to vehicle control. Enrichment analysis of differentially expressed genes (**B**) reveals the top pathway identified: ‘Metabolism of steroids’, which contains the genes AKR1C2, SCD, MVD, SREBF1 and FASN.

Because fibrogenic gene expression in phHSCs was significantly down-regulated by 30 nM of AZD3355 at 72 h (Fig. 2B), we selected this condition to perform transcriptomic analysis in order to more broadly characterize genes regulated by AZD3355. Twenty two genes were differentially expressed (P-adj < 0.1) between AZD3355 treatment and vehicle groups (Fig. 3A). Included in the up-regulated genes are STAR-related lipid transfer domain containing 9 (STARD9); sterol regulatory element binding transcription factor 1 (SREBF1); and Kielin cysteine rich BMP Regulator (KCP). Cancer-related transcripts of AKR1C2, TPT1 and RPS27A were also down-regulated by AZD3355 in phHSCs (Fig. 3A). We evaluated differential enrichment to establish if there were biological pathways enriched within this gene list. The top pathways identified were ‘metabolism of steroids’, ‘activation of gene expression by SREBF’ and ‘activation of gene expression by SREBP’(Fig. 3B).; the findings are interesting given the stellate cell’s important role in storing lipids within retinyl esters^23^, but were not further pursued.

We next identified the cell-signaling networks associated with these genes using the computational tool eXpression2Kinases (X2K)^24^. Transcription factor enrichment analysis revealed MYC as the top predicted transcription factor most likely regulating the observed changes in mRNA expression (Supplemental Figure S3A). The protein–protein interaction expansion analysis using the Genes2Networks algorithm identified the sub-network of connected transcription factors that regulated these genes (Supplemental Fig S3B). Kinase enrichment analysis prioritized the protein kinases known to phosphorylate substrates within the subnetwork of transcription factors and the intermediate proteins that connect them. MAP and ERK kinases were among the top kinases identified (Supplemental Fig S3C). Together these data was used to generate the network (Supplemental Fig S3D).

### AZD3355 inhibits *COL1a1, aSMA, TNFa* mRNAs and secreted Col1A1 in diseased human liver slices

**Figure 4:**
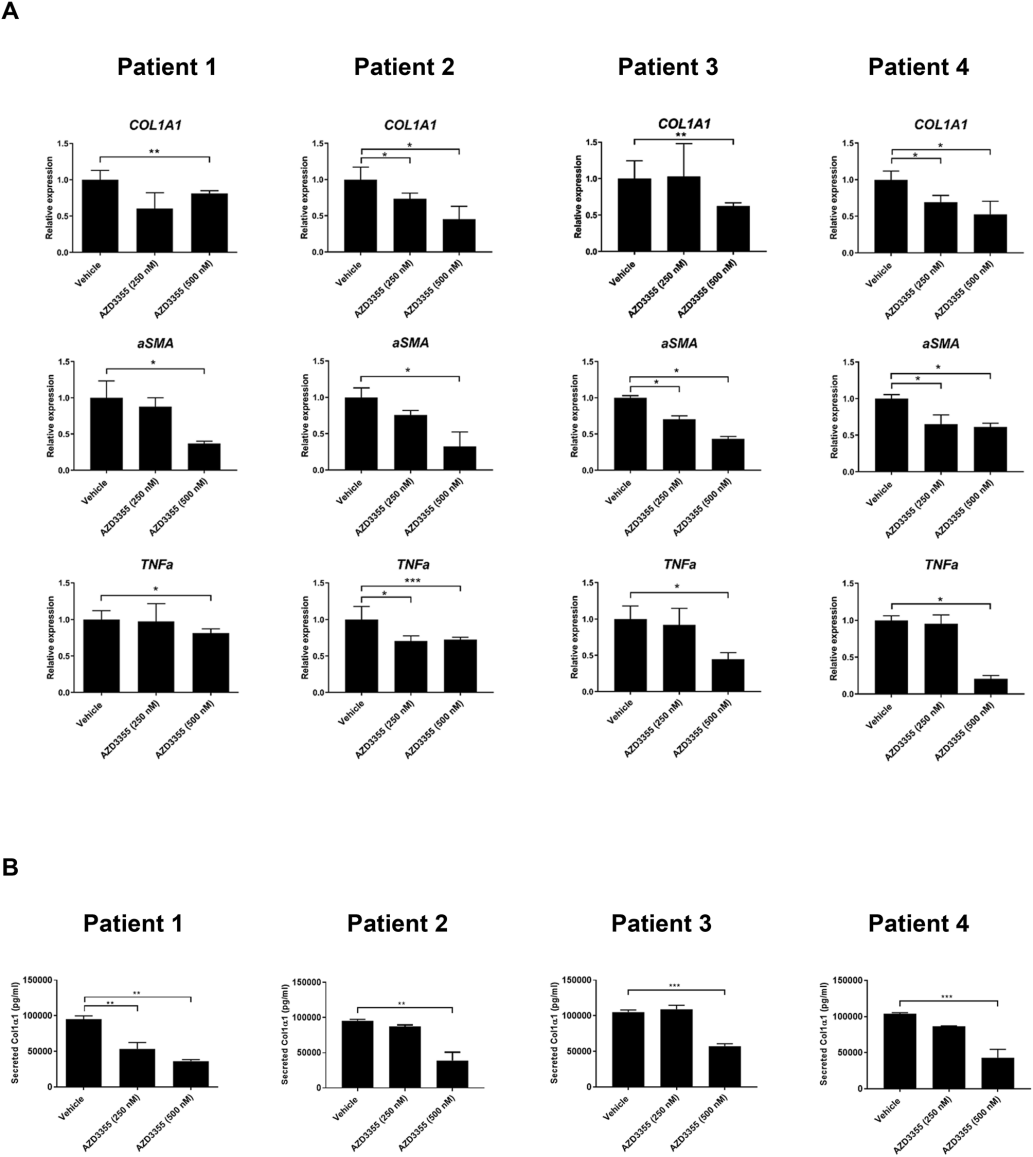
AZD3355 reduces fibrogenic and inflammatory gene expression as well as Col1α1 protein secretion in human liver slices. Precision cut human liver slices (hPCLS) from four different patient donor liver samples were cultured with either DMSO (as vehicle) or AZD3355 (250 and 500 nM) for 24 h. Quantitative PCR was used to measure mRNA expression for *Col1A1*, *aSMA* and *TNFa*. Two liver slices (8 mm diameter each) were used for each patient/drug concentration. *Col1A1*, *aSMA* and *TNFa* gene expression was significantly down-regulated by AZD3355 treatment in each patient samples (**A**). Secreted Col1α1 protein in culture media was significantly reduced compared to vehicle group by AZD3355 treatment in all four patient liver slices measured by ELISA (**B**). Results were expressed as means ± SEM (n = 3). *p < 0.05, **p < 0.01, ***p < 0.001.

To assess the effects of AZD3355 on pro-fibrotic and inflammatory response genes in a more physiological context, we used the precision cut human liver slice model (PCLS), which preserves all cell types within their native architecture to uniquely capture endogenous cell–cell interactions^25^. PCLS from de-identified resection samples of injured human liver were used to test the activity of AZD3355 (250 nM and 500 nM) or DMSO (negative control) following 24 h incubation. Four liver samples were used, 2 male and 2 female, with ages ranging from 38 to 74 years with different etiologies and fibrosis stages, including two NASH liver (Table 1). After 24 h, there was dose-dependent, significant down-regulation of key fibrogenic genes *COL1A1* and *aSMA* as well as the inflammatory response gene *TNFa* by AZD3355 in all four patient liver slices (Fig. 4A). ELISA was used to examine the effect on secreted Col1α1 of AZD3355. There was significant reduction of secreted Col1α1 by AZD3355 treatment compared to vehicle in all four slice donors consistent with effects on of *COL1A1* mRNA (Fig. 4B). There was no cytotoxicity from AZD3355 based on lack of LDH release in the culture media (Supplemental Figure S4).

**Table 1:** Characteristics of human liver donors for PCLS studies.

Patient #	Sex	Age (Years)	Diseases Background	Pathological Diagnosis	Fibrosis Stage
1	Female	63	Hilar cholangiocarcinoma (Non-viral)	Periductal fibrosis with extensive ductular reaction associated large duct obstruction. Acute and chronic cholangitis	F0
2	Female	74	Colorectal liver metastasis (NASH; HCV infected)	Moderately differentiated metastatic adenocarcinoma with colorectal primary. Steatohepatitis (Grade1)	F4
3	Male	60	Fibrolamellar HCC (Non-viral)	Moderately differentiated hepatocellular carcinoma. Noncirrhotic with mild macrovesicular steatosis	F0
4	Male	38	HCC (NASH; HBV infected)	Moderately differentiated hepatocellular carcinoma. Chronic Hep B infection with macrovesicular steatohepatitis	F2

### Effect of AZD3355 in a murine NASH model in vivo

We have previously established a murine model that closely resembles human NASH following 12 or 24 weeks of a high fat, high fructose, high cholesterol (‘Western’) diet with low dose CCl_4_ administration IP^26,27^. The model faithfully replicates both the progression of disease histologically from fibrosis to cancer, the transcriptomic features and metagenome of human NASH^26^; we now refer to this model as the FAT-NASH (**F**ibrosis **A**nd **T**umors, NASH) model. In the FAT-NASH model, AZD3355 (10 mg/kg or 30 mg/kg) was administered by gavage twice daily, 5 days per week, beginning at week 13 and continued for the remaining 12 weeks of the NASH protocol.

In mice administered AZD3355 compared to the vehicle group with NASH, the average body weight was significantly decreased by the 30 mg/kg dose, but not 10 mg/kg, beginning in week 2 of treatment (week 14 of the study) in males, and from week 3 (week 15 of the study) in females (Supplemental Figure S5A). The reduced body weight was not due to the toxic effect of AZD3355 dosing, as no significant differences in food or water intake were observed before and during AZD3355 administration (Supplemental Fig S5B and S5C).

AZD3355 also dose-dependently reduced hepatomegaly in both male and female mice. At 30 mg/kg AZD3355, the average liver weight and liver-to-body weight ratio were significantly decreased compared to vehicle-treated animals (Supplemental Fig S6A and S6B). Necro-inflammatory activity was assessed in part by serum aspartate aminotransferase (AST) and alanine aminotransferase (ALT) levels. Both in male and female mice the average AST and ALT, and in female mice, average triglyceride levels were significantly reduced (Supplemental Fig S6C and S6D). Average triglyceride trended towards reduction in male mice in response to AZD3355 compared to vehicle (Supplemental Fig S6C).

The NAFLD activity score (NAS) was assessed blindly according to NASH-CRN criteria by an experienced liver pathologist^28^. In male mice receiving vehicle, the NAS was 7.4 ± 0.2, which was reduced to 6.6 ± 0.6 in treated with 30 mg/kg AZD3355. In mice treated with 30 mg/kg OCA, NAS scores were reduced to 4.7 ± 0.7 in male and 4.7 ± 0.6 in female mice (Supplemental Fig S7B and 7C, Table 2). 30 mg/kg AZD3355 treatment as well as OCA also significantly reduced fibrosis stages in both male and female FAT-NASH mice (Figure S7B and S7C).

**Table 2:** NAFLD activity score (NAS) and fibrosis stage of AZD3355 treated mice.

Treatment	Male				Female			
	Vehicle	AZD3355 (10 mg/kg)	AZD3355 (30 mg/kg)	OCA (30 mg/kg)	Vehicle	AZD3355 (10 mg/kg)	AZD3355 (30 mg/kg)	OCA (30 mg/kg)
Steatosis	2.8 ± 0.1	2.6 ± 0.2	2.3 ± 0.2	2.0 ± 0.2	2.5 ± 0.1	2.5 ± 0.1	2.2 ± 0.1	2.0 ± 0.2
Hepatocyte Ballooning	1.7 ± 0.1	1.6 ± 0.1	1.7 ± 0.1	1.0 ± 0.2	1.2 ± 0.1	1.2 ± 0.1	1.2 ± 0.1	1.1 ± 0.2
Lobular Inflammation	2.7 ± 0.1	2.7 ± 0.1	2.5 ± 0.2	1.7 ± 0.2	2.3 ± 0.1	2.5 ± 0.1	2.7 ± 0.1	1.6 ± 0.1
NAS	7.4 ± 0.2	7.1 ± 0.4	6.6 ± 0.6	4.7 ± 0.7	6.1 ± 0.4	6.4 ± 0.4	6.4 ± 0.3	4.7 ± 0.6
Fibrosis stage	3.4 ± 0.1	3.1 ± 0.1	3.0 ± 0.0	2.7 ± 0.2	3.6 ± 0.1	3.0 ± 0.1	3.1 ± 0.1	2.2 ± 0.1

### Antifibrotic activity of AZD3355 in murine NASH

**Figure 5:**
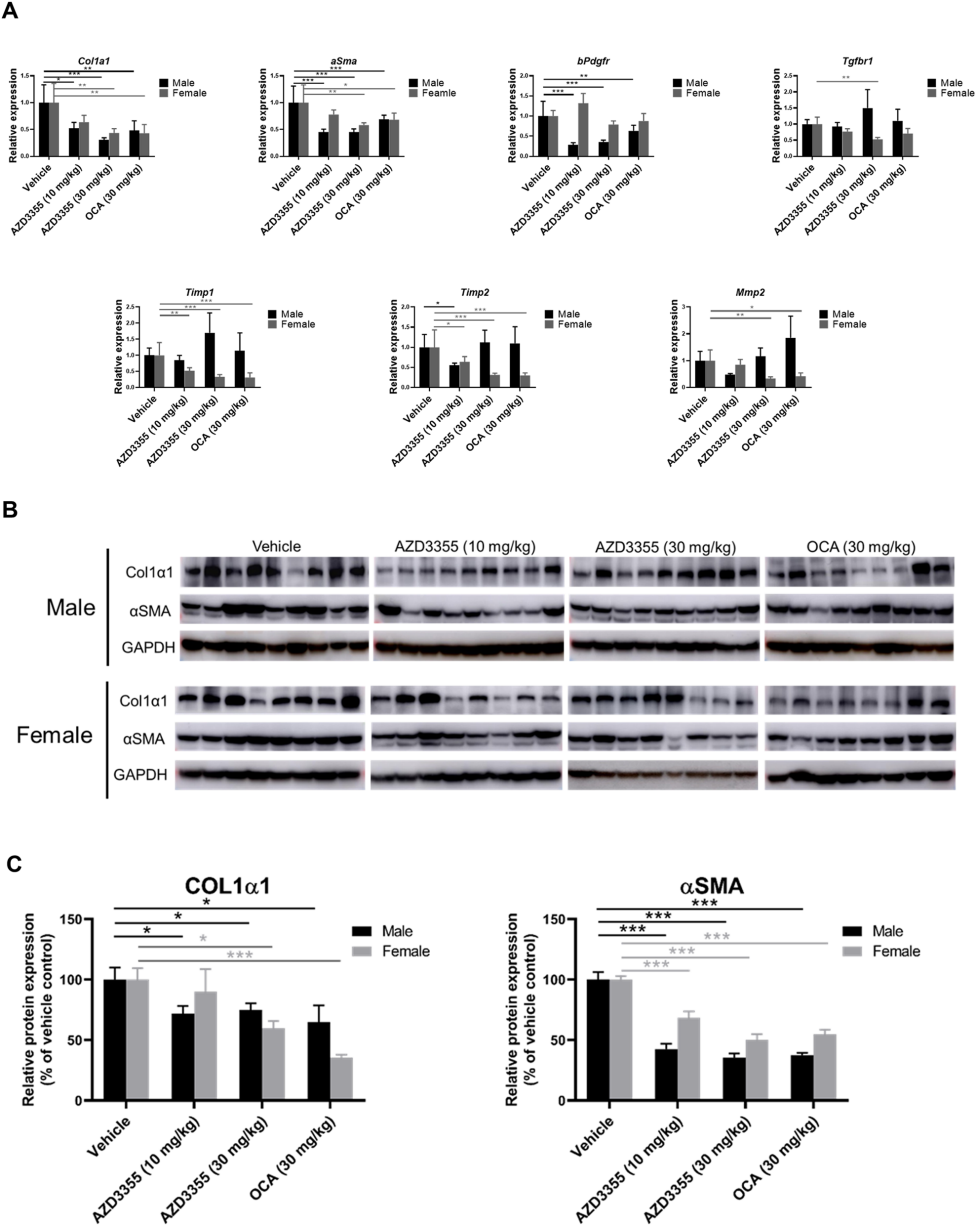
Expression of hepatic fibrogenic genes and proteins are down-regulated by AZD3355 in a murine NASH model. (**A**) Expression of fibrogenic mRNAs in male (black bar) or female (gray bar) murine liver tissues was quantified by RT-qPCR. Data was normalized to *GAPDH* and expressed relative to livers from the vehicle-treated group. (**B**) Western blots for Col1α1, αSMA and GAPDH in male and female mice from whole liver lysates. (**C**) Relative protein expression compared to the vehicle-treated group was quantified by densitometry of respective Western blots bands, and GAPDH was used as internal control. Vehicle: n = 9 male, 8 female; AZD3355 (10 mg/kg): n = 9 male, 8 female; AZD3355 (30 mg/kg): n = 9 male, 8 female; OCA (30 mg/kg): n = 9 male, 8 female animals. Results were expressed as means ± SEM. *p < 0.05, **p < 0.01, ***p < 0.001.

We also assessed the effect of AZD3355 on murine NASH by quantifying whole liver mRNA and protein expression for key markers of hepatic fibrogenesis. There was a dose-dependent down-regulation of mRNAs for *Col1α1*, *αSma*, and *Pdgf-Rβ* in both male and female animals compared to vehicle (Fig. 5A). Interestingly, gene expression for *TGFβ-R1, TIMP1* and *MMP2* was down-regulated in female mice only (Fig. 5A). In mice treated with OCA, *Col1α1*and *αSma* gene expression were down-regulated in both male and female mice, whereas *Pdgf-Rβ* was reduced in male mice; *TIMP1, TIMP2* and *MMP2* were reduced in female mice (Fig. 5A).

We also measured expression of Col1α1 and αSma by Western Blot from whole liver lysates (Fig. 5B,C). Both Col1α1 and αSMA proteins were reduced in male mice administered either 10 or 30 mg/kg AZD3355 (Fig. 5C). In female mice, αSMA protein expression was reduced with both 10 and 30 mg/kg AZD3355, however Col1α1 protein expression was down-regulated in females treated with AZD3355 30 mg/kg (Fig. 5C). In OCA treated animals Col1α1 and αSMA protein were significantly down-regulated in both male and female mice (Fig. 5C).

### AZD3355 reduces collagen content in a murine NASH model

**Figure 6:**
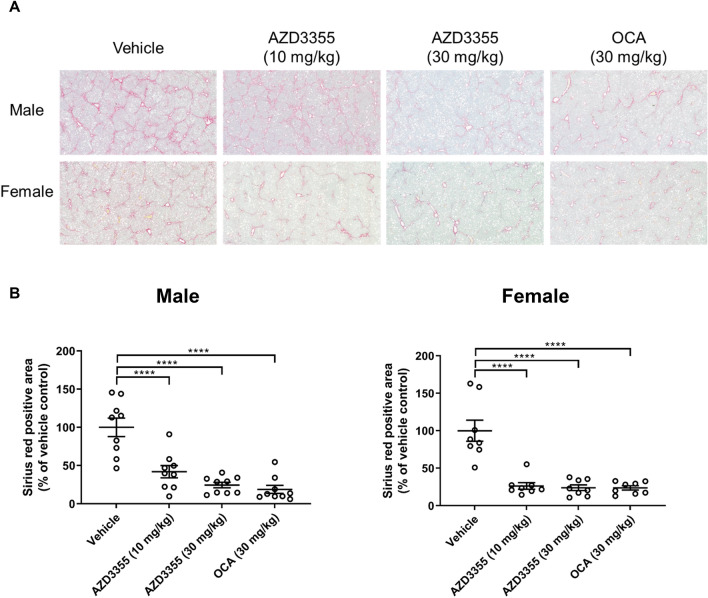
Hepatic collagen deposition is reduced by AZD3355 treatment in a murine NASH model. Representative photomicrographs (20 X magnification) of Sirius Red stained liver sections (**A**) from male and female mice treated with either vehicle or AZD3355 (10 and 30 mg/kg) and OCA (30 mg/kg). Morphometric quantification of Sirius Red-positive area (**B**) was performed using BIOQUANT image analysis software. Dose dependent reduction of hepatic fibrillar collagen formation by AZD3355 treatment in both male and female mice was assesed. Vehicle: n = 9 male, 8 female; AZD3355 (10 mg/kg): n = 9 male, 8 female; AZD3355 (30 mg/kg): n = 9 males, 8 females ; OCA (30 mg/kg): n = 9 male, 8 female animals. Results were expressed as means ± SEM.*p < 0.05, **p < 0.01, ***p < 0.001.

**Figure 7:**
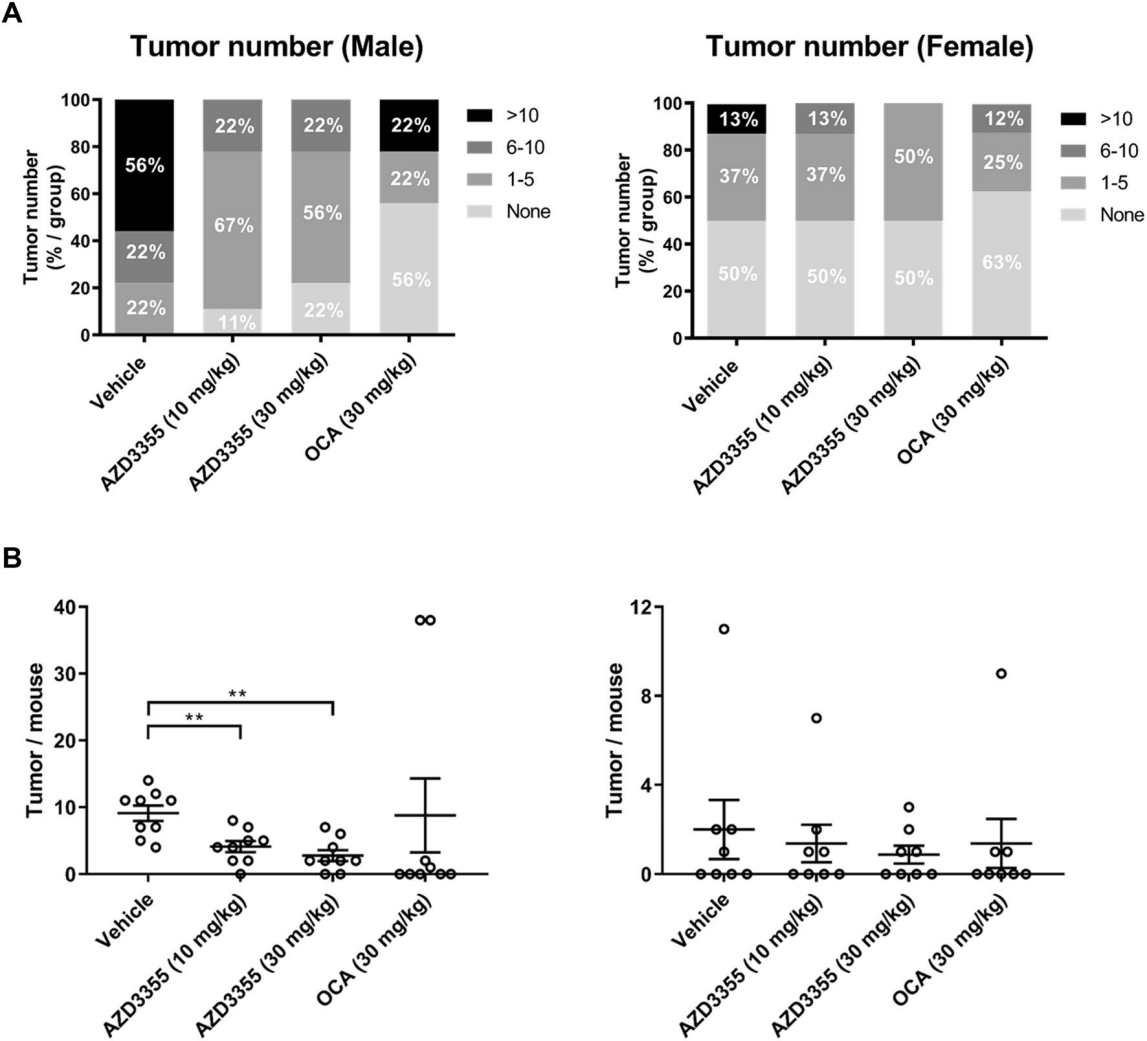
Reduced tumor development by AZD3355 treatment in murine NASH. Reduced tumor development in male and female mice was evident in AZD3355-treated mice compared to NASH mice treated with either vehicle or obeticholic acid (OCA) (30 mg/kg). As expected, tumor numbers at 24 weeks were greater in males than females in NASH mice treated with vehicle (100% in male and 50% in female), but both were reduced by AZD3355 treatment (**A**). Dose dependent reduction of total number of HCC per mouse by AZD3355 treatment was established (**B**). Vehicle: n = 9 males, 8 females; AZD3355 (10 mg/kg): n = 9 males, 8 females; AZD3355 (30 mg/kg): n = 9 males, 8 females; OCA (30 mg/kg): n = 9 males, 8 female animals. Results were expressed as means ± SEM. *p < 0.05, **p < 0.01, ***p < 0.001.

To assess hepatic fibrosis in murine NASH, collagen accumulation was quantified by morphometry of picrosirius red-stained liver sections from vehicle and drug-treated mice. Severe bridging fibrosis was present in both male and female mice administered vehicle only, which was reduced in AZD3355-treated animals (Fig. 6A). By morphometry of Sirius Red stained tissue collagen content was significantly reduced in both male and female animals by 10 or 30 mg/kg AZD3355, (Fig. 6B). This results are consistent with the pathological scoring of fibrosis (Figure S7B and S7C). Collagen deposition in obeticholic acid- treated control mice was also significantly reduced in both male and female groups (Fig. 7B).

### Reduced hepatocellular carcinoma development in FAT NASH mice treated with AZD3355

A consistent feature of this FAT-NASH model is the development of hepatocellular carcinoma (HCC) in all male animals at 24 weeks^26^. Similarly, in the current study there was robust tumor development with vascular invasion in some animals (Supplemental Figure S8). In males, 100% developed HCC in the vehicle-treated group. In females treated with vehicle, 50% developed HCC (Fig. 7A). AZD3355 treatment of male mice led to a dose-dependent reduction of tumor development; 11% and 22% mice did not develop HCC by AZD3355 10 mg/kg and 30 mg/kg, respectively and the total number of tumors was significantly reduced (Fig. 7A). In OCA treated animals the number of tumors was reduced in both male and female mice.

## Discussion

In this study we have computationally identified AZD3355 as a potential therapeutic for NASH and have experimentally validated this in culture, ex vivo and using an in vivo model. AZD3355 was initially designed to be a potent, peripherally-restricted gamma-aminobutyric acid receptor B (GABA-B) receptor agonist with a preclinical therapeutic window to inhibit transient lower esophageal sphincter relaxations (TSLERs) with over 40X greater potency compared to a standard therapy, Baclofen^29,30^. However, Phase 2b testing indicated that AZD3355 did not achieve a clinically meaningful effect on GERD outcomes^19,30,31^, and further development in this indication was no longer pursued by AstraZeneca Pharmaceuticals.

Our data in these NASH-related studies demonstrate that the compound, a GABA-B receptor agonist, has antifibrotic, anti-inflammatory, and hepatoprotective activity in cultured primary and immortalized human stellate cells, in human liver slices, and in a murine model of NASH whose features closely mirror human NASH. These findings reinforce the promise of computational based drug repurposing to more rapidly identify therapeutic candidates, an approach that has been validated in other diseases such as inflammatory bowel disease^14^ and cancer^13^, but not previously in NASH. Thus, our findings add to a growing body of evidence validating the reliance on unbiased data representations of complex molecular networks that underly disease to uncover novel pathogenic pathways or therapeutic targets.

The direct reduction in fibrosis by AZD3355 in cultured primary and immortalized human HSCs and in vivo in mouse reveals a novel intracellular pathway of gene regulation in this cell type, the principal fibrogenic cell in liver injury following its activation into proliferative myofibroblasts^32,33^. The finding is consistent with an earlier study describing effects of GABA-B receptor agonism on fibrosis and αSMA expression (a classical marker of HSC activation) in cultured rat stellate cells, and in vivo in CCl_4_ injury in rats^34^. Interestingly, both that study and our own data confirm a direct antifibrotic activity but no effect of GABA-B agonism on stellate cell proliferation. GABA-B receptor agonism also inhibited TGFβ1 secretion in the previous publication^34^, which could account for its antifibrotic effect in our studies as well; although we did not measure this cytokine directly, we documented reduced TGFβ1 gene expression in vivo. Interestingly, we identify Myc as the top transcription factor regulated in cultured HSCs in response to AZD3355 which indicates it may play a central role in regulating the observed changes in mRNA expression; MYC which has previously been implicated in HSC activation^35,36^, but has never been fully explored in this cell type. Since inactivating oncogenic MYC is a major target of cancer therapies^37^, this finding raises the prospect of further defining the role of MYC as a direct antifibrotic target in liver fibrosis as well. Interestingly, AZD3355 reduced expression of several other liver HCC-related genes in HSCs, AKR1C2^38^, TPT1^39^, RPS15A^40^ and RPS27A^41^. While HSCs are not the cell of origin in HCC, reduced expression of these genes in response to AZD3355 might alter their potential to promote tumors in NASH, as stromal gene expression can be an important determinant of HCC outcomes^42,43^.

Our findings also suggest anti-inflammatory and hepatoprotective activities of AZD3355 as well based on reduced *TNF-*α mRNA in human liver slices, and reduced serum AST and ALT in vivo in the NASH model, respectively. These effects may indirectly further reduce HSC activation and fibrogenesis, but more mechanistic studies are warranted to firmly establish this possibility. Nonetheless, the multiple direct and indirect benefits of AZD3355 reinforce its appeal as a potential therapy for NASH. To date, the most advanced NASH therapy in phase 3 trials is obeticholic acid, which like AZD3355, has multiple targets of activity in the disease^7,44,45^; the drug showed efficacy in a phase 3 trial^7^ but is not yet approved. Here we further establish the therapeutic effect of obeticholic acid as a positive control in our study, which has comparable activity to AZD3355. The rising number of drug failures in NASH^46,47^ by agents that have a single cellular or molecular target indicate that agents with multiple targets such as AZD3355 may be more promising.

The sex difference in the extent of injury following AZD3355 is an additional finding of interest, with lower AST/ALT in female mice associated with decreased α-SMA and collagen expression in vivo, as well as greater liver cancer development in males. While a greater propensity towards liver cancer in male mice and humans is well described and thought to reflect different regulatory pathways^48^, sex differences in human NASH have not been as well characterized^49–51^. Nonetheless, sex is a significant factor in determining patterns of gene expression in human NASH^49^, and further studies addressing gender as a determinant of NASH risk and disease in animal models are needed.

The heterogeneous responses to AZD3355 among human precision-cut liver slices from different donors is also a noteworthy observation, and may mirror the challenges of heterogeneity in clinical trials as well. While reliance on inbred mouse strains or disease modeling provides more interpretable readouts of drug efficacy, these models overestimate drug activity following their translation into human trials. This has been especially true in NASH, where in vivo modeling in rodents typically yield more optimistic results than is subsequently seen in human clinical trials. Thus, greater reliance on systems such as PCLS that reflect real-world heterogeneity may be highly informative in predicting clinical success, and could provide insights into the factors (i.e. genetic, epigenetic or even metagenomic) that contribute to this heterogeneity.

These results provide further validation of data-driven, hypothesis-free approaches to uncover novel therapies using existing drugs with established safety profiles^16,52^. In the example of AZD3355, the agent had no apparent link to fibrotic liver disease, yet transcriptomic profiling uncovered its potential efficacy, which we have validated in this study. Moreover, since the drug’s mechanism of action includes potent direct antifibrotic activity towards fibrogenic cells, it may be active in other chronic as well as fibrotic diseases in liver and other tissues.

## Materials and methods

### Computational drug repurposing method to identify candidate NASH therapies:

We used a modified connectivity mapping approach to identify potential novel use cases for a collection of 32 compounds, each of which had completed Phase 1 testing or greater (https://openinnovation.astrazeneca.com/data-library.html#transcriptomicprofilingdata). Compound signatures were obtained by exposing cell lines (A549 and MCF7, from ATCC^53^) to each compound, at a high and low dose, and generating a RNASeq signature, thus resulting in four separate signatures for each compound (e.g. “Compound-1 A549 High dose”, “Compound-1 MCF7 High dose”, “Compound-1 A549 Low dose”, “Compound-1 MCF7 Low dose”). These were then compared to a disease library (generated from publicly available data^20,21^) and a connectivity score was obtained for each compound-condition/disease pair^54^. This approach has previously been described by us and others^20,52^. Compound-condition/disease connectivity scores, that were significantly (FDR < 0.01) negative (connectivity score < 0), indicate that under that particular cell-dosage combination, the compound is predicted to “normalize” the disease signature towards the transcriptomic profile observed in healthy controls. The NASH signature used in the disease mapping step was generated from liver biopsies collected from 12 NASH patients, and five control subjects ^55,56^ available from Gene Expression Omnibus (Accession: GSE24807).

### Gene set enrichment analysis of AZD3355 against human NAFLD data sets

We generated AZD3355 gene expression perturbation signatures with up- and down-regulated genes in the A549 and MCF7 cell lines with a false discovery rate cutoff of p < 0.05. We then used these gene sets as inputs for a Gene Set Enrichment Analysis^57^ to quantify enrichments of these AZD3355 signatures in human NAFLD datasets available from Gene Expression Omnibus (see Fig. 1E for references).

### Analysis of AZD3355 effects in immortalized (LX-2) and primary human stellate cells (phHSCs)

AZD3355 (FW 141.08) was supplied by AstraZeneca Pharmaceuticals to purity > 99% by HPLC. The development and structure of AZD3355 has been previously described^58^. For cell culture studies, AZD3355 was reconstituted in normal saline (0.9% sodium chloride) at 2 mM concentration stock solution followed by a series of working concentrations of 1, 3, 10, 30, 100 and 300 nM in DMEM cell culture medium (Thermo Fisher Scientific, MA), supplemented with 0.1% BSA. Both stock and working solutions were made fresh before each experiment. As a positive control the cells were treated in parallel with sorafenib (LC laboratories, MA) at 7,500 nM concentration dissolved in sterile DMSO, since this drug has antifibrotic activity in cultured stellate cells^59^.

The activity of AZD3355 was analyzed in LX-2 cells, an immortalized human hepatic stellate cell line^22^ and primary human hepatic stellate cells (phHSCs) isolated from discarded remnants of surgically resected human livers^60^, which lacked patient identifiers. Use of these de-identified tissues to generate phHSCs was approved by the Institutional Review Board (IRB) at the Icahn School of Medicine at Mount Sinai, NY. Procedures of phHSC isolation, culture and their purity assessment by immunostaining has been described previously^60,61^. Prior to all drug incubations, both LX-2 cells and phHSCs were maintained overnight in starvation media comprised of DMEM supplied with 0.1% BSA (without antibiotics) to synchronize cell metabolic activities. The cells were incubated with incremental concentrations of AZD3355 or sorafenib for 24, 48 and 72 h.

### Cytotoxicity assay

5000 LX-2 cells or 10,000 phHSCs were plated per well in 96 well plates, serum-starved overnight, then incubated with either vehicle different concentrations of AZD3355 in starvation media for the indicated durations. MTS assays were performed using CellTiter 96 Aqueous One Solution Cell Proliferation Assay kit (Promega, WI) according to the manufacturer’s protocol.

### Cell proliferation assay

LX-2 cells (5000 cells per well) or phHSCs (10,000 cells per well) were plated in 96 well plates, serum-starved overnight, then incubated with incremental concentrations of AZD3355 in starvation media. After the end of drug incubation (24, 48 or 72 h), the cells were labeled with BrdU for either 2 h (for LX-2 cells) or 16 h (for phHSCs) at 37 °C in 5% CO_2_. Cell proliferation was quantified using the cell proliferation ELISA, BrdU colorimetric kit (Roche, NY) according to manufacturer’s instructions. Absorbance was measured at 370 nm with reference wavelength at 492 nm.

### Cell apoptosis assay

LX-2 cells (5000 cells per well) or phHSCs (10,000 cells per well) were plated in 96 well clear bottom black plates. After overnight serum starvation the cells were incubated with AZD3355 at the indicated concentration or 3% DMSO (as a positive control to induce apoptosis). After 72 h, the fluorescence signal of Caspase-3 and-7 activities were measured using a Synergy HT (BioTek Instrument Inc., VT) spectrofluorometer using Apo-ONE Homogeneous Caspase-3/7 Assay kit (Promega, WI) according to the manufacturer’s protocol.

### RT-quantitative PCR in human HSCs

LX-2 (150,000 cells) or phHSCs (200,000 per well) were plated in 6-well plates. After overnight serum starvation the cells were incubated with either vehicle, AZD3355 or sorafenib at the indicated concentrations for 48 or 72 h. For LX-2 cells the vehicle and the drug were replaced after 48 or 72 h by normal DMEM with 10% FBS, then maintained for an additional 48 or 72 h, respectively. Cells were harvested and total RNA was extracted using RNeasy Mini Kit (Qiagen, CA) according to manufacturer protocols. 0.5 µg of total RNA was used for reverse transcription with ‘RNA to cDNA EcoDry Premix (Double Primed) Kit’ (Clontech, CA). Expression of fibrogenic genes was quantified by qPCR using custom designed primers (Sigma-Aldrich, MO) and iQ SYBR Green Supermix (Bio-Rad, CA) on a LightCycler 480 II (Roche Diagnostics Corporation, IN). Glyceraldehyde-3-phosphate dehydrogenase (GAPDH) were used as housekeeping gene to determine the relative expression of fibrogenic genes.

### RNA sequencing analysis of primary human hepatic stellate cells (phHSCs)

Two hundred thousand phHSCs per well were plated in 6-well plates. After overnight serum starvation the cells were treated with either vehicle or 30 nM of AZD3355 in DMEM with 0.1% BSA for 72 h. Cells were harvested and total RNA was extracted using the RNeasy Mini Kit as described in the RT-quantitative PCR method. The purity of RNA (RNA integrity number or RIN) was assessed using 2100 Bioanalyzer instrument (Agilent, CA). To prepare a high quality sequencing library from mRNA we used the TruSeq Stranded mRNA library prep kit (Illumina, CA) according to manufacturer's protocol. Sequencing was performed on NextSeq Hi (Illumnina, CA) sequencing instrument with setting at following output: R1 = 100; index = 8; R2 = 100. RNASeq raw data were processed using FAST QC software (http://www.bioinformatics.babraham.ac.uk/projects/fastqc/) and aligned with STAR aligner^62^ via a computational pipeline implemented in NextFlow^63^. After alignment and summarization with featureCounts, data was normalized and differential expression was carried out with DESeq2^64^. DEGs were considered significant at FDR < 0.1. Annotation of enriched gene pathways was performed using ClusterProfiler^65^. Genes identified from the RNASeq were uploaded into the eXpression2Kinases (X2K) web tool^24^ a pipeline consisting of transcription factor enrichment analysis, protein–protein interaction network generation, and kinase enrichment analysis. Principle Components Analysis (PCA) was performed on counts after applying a variance-stabilizing transformation and summarized using pcaExplorer^66^. RNASeq data generated from phHSCs is available at GSE178703.

### Human precision-cut liver slices

Human precision-cut liver slices (hPCLS) were generated from discarded remnants of surgically resected human livers^67^ that lacked patient identifiers, following Institutional Review Board (IRB) approval at Icahn School of Medicine at Mount Sinai. Four different patient backgrounds for liver samples were selected for the hPCLS study, of which 2 were male, 2 were female (Table 1). Normal-appearing resection margins surrounding HCC or metastatic regions were selected for hPCLS generation. The ischemic time between post-hepatectomy and generated PCLS was 3–4 h. The resected liver pieces were transported to the lab in ice-cold Krebs–Henseleit buffer. Cores of 8 mm diameter were generated by using a stainless-steel coring tool (Alabama Research and Development, AL), and the liver core was attached to the specimen holder by solvent- free cyanoacrylate adhesive (Best Klebstoffe GmbH & Co. Germany). The liver core attached to the specimen holder was mounted in the buffer tray, which was submerged in carbogen saturated ice-cold Krebs–Henseleit buffer supplemented with 25 mM glucose, and the liver slices were created using VT1200S tissue slicer (Leica Biosystem, IL). During slicing Krebs–Henseleit buffer was continuously supplied with carbogen. Intact liver slices (200 µm thickness) were collected from the buffer tray using a soft bristle brush and transferred to six well tissue culture plate (3 slices/well) filled with 37 °C warm William’s E GlutaMAX (WE-GlutaMAX) media (Thermo Fisher Scientific, MA) supplemented with 25 mM glucose and 50 µg/ml gentamycin (Thermo Fisher Scientific, MA). The slices were pre-incubated in a 37° C incubator supplemented with 5% CO_2_ and 95% O_2_ on a gentle rocker (10 rpm) for four hours to equilibrate the tissues. After four hours pre-incubation (restoration period of liver slices), fresh medium was added containing either vehicle (DMSO) or two different concentration of AZD3355 for additional 24 h. At the end of drug treatment from each condition, slices were used for total mRNA extraction. Collagen1a1 (*COL1A1*), alpha smooth muscle actin (*aSMA*) and tumor necrosis factor alpha (*TNFa*) were quantified using RT-quantitative PCR. For assessment of secreted Col1α1, culture media from each condition were collected, snap frozen and measured by ELISA as described previously^59^. For cytotoxicity assessment the luminescence signal of secreted lactate dehydrogenase (LDH) in media was measured using a Synergy HT (BioTek Instrument Inc., VT) luminometer using LDH-Glo cytotoxicity assay kit (Promega, WI) according to the instructions and purified lactate dehydrogenase from rabbit muscle as standard or positive control.

### In vivo assessment of AZD3355 efficacy in a murine NASH model

The animal protocol was approved by the Institutional Animal Care and Use Committee (IACUC) at the Icahn School of Medicine at Mount Sinai, NY (IACUC-2018-0060). Six week old male and female C57BL/6J mice (housed separately) were purchased from Jackson Laboratories (Farmington, CT). Five mice per cage were housed in a Helicobacter-free room for 12 h light–12 h dark cycle and weighed once weekly.

Carbon tetrachloride (CCl_4_) was purchased from Sigma-Aldrich, MO. CCl_4_ was freshly dissolved in corn oil at final concentration of 5% before injection. The final dose of pure CCl_4_ was 0.2 µl/g of body weight of mice, delivered intraperitoneally once/week starting from initiation of the western diet/sugar water feeding and continued for a total period of 24 weeks. Western diet containing 21.2% fat (42% Kcal), 41% sucrose and 1.25% cholesterol by weight was purchased from Envigo, WI (Teklad Custom diet). Sugar water solution contained 18.9 g/L D-( +)-Glucose (Sigma-Aldrich, MO) and 23.1 g/L D-(−)-Fructose (Sigma-Aldrich, MO) dissolved in autoclaved water and filter sterilized. The diet and sugar water were replaced twice weekly.

Methylcellulose (4,000 cP) (Sigma-Aldrich, MO) diluted in UltraPure Distilled water (ThermoFisher Scientific) to 0.5% w/v and maintained at 4 °C, was used as a drug delivery vehicle. Two different concentrations of AZD3355 (2 mg/ml (w/v) dilution for 10 mg/kg and 6 mg/ml (w/v) dilution for 30 mg/kg dosing) were made in 0.5% methylcellulose before each gavage. Obeticholic acid (OCA; FW 420.63) was used as a positive control, purchased from ApexBio (Houston, TX). OCA was also made fresh in 0.5% methylcellulose (6 mg/ml (w/v); OCA dilution for 30 mg/kg dosing) each week, aliquoted and stored at −20 °C.

The experimental scheme of AZD3355 treatment in the FAT-NASH model is summarized in Supplemental Figure S1. Both male and female C57BL/6J mice were fed a Western diet and sugar water, combined with weekly CCl_4_ injection via IP for 24 weeks. At week 12, five mice from each sex were sacrificed and NASH activity was assessed by: i) liver enzyme and lipid panel analysis in blood serum level, ii) pro-fibrotic gene and protein expression, iii) hepatic collagen quantification and iv) NAFLD activity scoring (data not shown). Beginning at week 13, the mice were randomly divided into five groups (9 males and 8 females per group) and treated for an additional 12 weeks as follows: i) 0.5% methylcellulose as vehicle, ii) AZD3355 at 10 mg/kg body weight, iii) AZD3355 at 30 mg/kg body weight, iv) Obeticholic acid at 30 mg/kg body weight (as positive treatment of NASH) and v) NASH background disease-control mice (5 mice)—only diet and CCl_4_ without vehicle or drug (group v data are not shown). The mice were dosed BID in 12-h intervals for vehicle and AZD3355 (5 days/week) and QD for OCA (5 days/week) via gavage. Weekly body weight, WD and sugar water consumption were also recorded in each group. At the end of the experiment the animals from each group were sacrificed, with blood and liver collected for analyses.

At the termination of the study, blood samples were collected through the inferior vena cava for serum preparation. The liver and spleen were excised, cleaned in 1X PBS, and weights recorded. A small piece from the left liver lobe of each animal was excised and preserved in RNAlater (Qiagen, MD) for mRNA quantification. Another small piece from the left lobe was snap-frozen in liquid nitrogen for protein extraction. A third small piece from the left lobe was fixed in 10% formalin.

### Blood serum preparation, liver enzymes and lipid panel analysis

For blood serum preparation collected blood samples were kept at room temperature for 30 min to allow clotting. Serum was collected (top layer) after centrifugation at 2000×*g* for 10 min at 4 °C. Aspartate aminotransferase (AST), alanine aminotransferase (ALT) and triglycerides were measured from serum using an ARCHITECT c16000 Clinical Chemistry Analyzer (Abbott Diagnostics, MA) in the Mount Sinai Clinical Chemistry Laboratory facility according to the manufacturer’s instruction.

### Picrosirius red staining for collagen quantification in liver sections

Liver was fixed in 10% formalin buffer and paraffin embedded liver tissues were sectioned using a 4 µm microtome. Slides containing tissue sections were baked at 60 °C for 1 h and re-hydrated through xylene followed by graded ethanol (100%, 95%, 85% and 70%) into distilled water and processed for picrosirius red/fast green staining. For collagen staining, re-hydrated slides were stained for one hour in saturated picric acid with 0.1% Sirius Red (Direct Red-80; Sigma-Aldrich, MO) followed by counterstain with 0.01% Fast Green (Sigma-Aldrich, MO) for another hour. The slides were removed from the stain, rinsed in water and rapidly dehydrated through graded ethanol (70%, 85%, 95% and 100%) followed by xylene and finally placed on cover slips in Permount (ThermoFisher Scientific, NJ). Whole slides with staining sections were digitally scanned in an Aperio AT2 digital scanner (Leica Biosystems Inc., IL). The image from each scanned section was randomly saved as 5× zoom level (3 images/section) in Aperio ImageScope histopathological diagnostic software [v12.4.0.5043] (Leica Biosystems Inc., IL). A total of 6 sections/animal were stained and 3 images from each section (total 18 pictures/animal) were evaluated using BIOQUANT image analysis software v16.5.60 (Bioquant Image Analysis Corporation, Nashville, TN, https://bioquant.com) to quantify collagen accumulation in liver tissue.

### H&E staining and histopathological scoring of liver sections

A total of 2 sections/animal were stained with H&E. Steatosis, hepatocyte ballooning, lobular inflammation, portal inflammation and fibrosis in liver sections were scored according to the NASH Clinical Research Network (NASH CRN) scoring system in a blinded fashion by an expert hepato-pathologist. NAFLD activity score (NAS), ranging from 0 to 8, was calculated^28^ based on the sum of scores of steatosis (0–3), hepatocyte ballooning (0–2) and lobular inflammation (0–3). Fibrosis (0–4) or portal inflammation (0–3) scores were assesed separately and not included in NAS.

### RT-quantitative PCR in liver tissue

Total mRNA was extracted using the RNeasy Mini Kit (Qiagen, CA). In brief, ~ 20 mg of liver tissue was lysed in 400 µl RLT-buffer combined with β-mercaptoethanol. Lysates were homogenized in the presence of 5 mm stainless steel beads (Qiagen, Germantown, MD) using a TissueLyser LT homogenizer (Qiagen, Germantown, MD) at 50 Hz/second for 2 min. The tissue lysate was passed through QIAshredder column (Qiagen, CA) by spinning the column at high speed for 1 min. The flow through was collected and mRNA was purified. RT-qPCR was performed as described above. Glyceraldehyde-3-phosphate dehydrogenase (GAPDH) was used as a housekeeping gene to normalize expression.

### Protein quantification in liver tissue

Total protein was extracted from ~ 20 mg of liver tissue using RIPA buffer (50 mM Tris–HCl pH8.0, 150 mM NaCl, 1% IGEPAL, 0.5% Sodium Deoxycholate and 0.1% SDS) along with Pierce Protease Inhibitor Mini Tablets, EDTA-Free (Thermo Scientific, IL) and phosphatase inhibitor Cocktail (Thermo Scientific, IL). Total protein was collected from the homogenate (middle aqueous phase) after centrifugation at 14,000 rpm for 10 min at 4 °C, and measured by Bradford colorimetric assay using Protein Assay Dye Reagent Concentrate (Bio-Rad, CA). 15 µg of proteins were loaded in NuPAGE 4–12% Bis–Tris gels (Thermo Scientific, IL). After transfer to the PVDF membrane the blot was blocked with 5% non-fat milk in 1X PBS. The primary antibodies were rabbit anti-Collagen1 (Bioss, MA), rabbit anti-αSMA (Abcam, MA) and mouse anti-GAPDH (Millipore, CA). After hybridization with HRP-conjugated secondary antibody (either Goat anti-rabbit HRP (Jackson ImmunoResearch Laboratories, PA) or anti-mouse IgG-HRP (Cell Signaling Technology, MA) the membrane was treated with Immobilon Western Chemiluminescent HRP substrate (Millipore, MA) and the signals were captured with Amersham Imager 6000 (GE Healthcare, PA). Protein bands of 210 kD for Col1α1 and 42 kD for αSMA were recognized by the respective antibodies. A 37 kD band of GAPDH was quantified as the loading control. For densitometric measurement of the protein bands, images were exported and analyzed using ImageJ 1.50f. software (http://imagej.nih.gov/ij) and bands were normalized to the loading control GAPDH.

### Statistical analysis

Data analysis was performed using GraphPad Prism v8.0.1 statistical software (GraphPad Software, Inc., San Diego, CA, http://www.graphpad.com). Standard error mean (± SEM) was calculated according to unpaired two tailed Mann–Whitney test where Gaussian distribution is non-parametric. Unless otherwise specified, *p* values < 0.05 were considered statistically significant vs vehicle group.

### Institutional approvals

All animal studies were approved by the Mount Sinai IACUC. Use of human tissues for precision cut-liver slices was IRB-exempt in accordance with guidelines of the Mount Sinai Institutional Review Board, as there were no patient-identifiers and the tissue was otherwise intended to be discarded.

All other methods were performed in accordance with the relevant institutional guidelines and regulations, and in accordance with ARRIVE guidelines^68^.

## ﻿Supplementary Information

Supplementary Information.
