# Supplementary material for: Repositioning of a novel GABA-B receptor agonist, AZD3355 (Lesogaberan), for the treatment of non-alcoholic steatohepatitis
Source: Sci Rep. 2021 Oct 21;11:20827. doi: 10.1038/s41598-021-99008-2 (PMC8531016; doi:10.1038/s41598-021-99008-2)
Supplement: Supplementary file 1 — Supplementary Information. [file 41598_2021_99008_MOESM1_ESM.docx]

**Repositioning of a Novel GABA-B receptor Agonist, AZD3355 (Lesogaberan),**

**for the Treatment of Non-Alcoholic Steatohepatitis**

Dipankar Bhattacharya^1^, Christine Becker^2^, Benjamin Readhead^2#^, Nicolas Goossens^1^^,

Jacqueline Novik^2^, Maria Isabel Fiel^3^, Leslie P. Cousens^4^, Björn Magnusson^5^, Anna Backmark^5^, Ryan Hicks^6^, Joel T. Dudley^2^ and Scott L. Friedman^1*^

**Supplemental Figure Legends**

**Figure S1. Schematic of experiments using the murine FAT-NASH model.** Six week old male and female C57BL/6J mice were maintained on Western diet and sugar water combined with CCl_4_ injection via IP per week (see Methods). At 12 week 5 of each male and female mice were sacrificed to determine the progression of disease of the model. Beginning at week 13 the mice were administered with either vehicle (0.5% Methylcellulose in water), AZD3355 (10 mg/kg and 30 mg/kg) or OCA (30 mg/kg) via gavage for next 12 weeks (from 13 to 24 weeks). At the end of the experiment (24 weeks) the animals from each group were sacrificed, with blood and liver collected for analysis. Vehicle: n = 9 male, 8 female; AZD3355 (10 mg/kg): n = 9 male, 8 female; AZD3355 (30 mg/kg): n = 9 male, 8 female; OCA (30 mg/kg): n = 9 male, 8 female animals. OCA, obeticholic acid; MC, Methylcellulose

**Figure S2. Hepatic stellate cells tolerate up to 100 nM AZD3355 for 72 hours.** (A) MTS cytotoxicity assay, BrdU cell proliferation assay and Caspase 3/7 apoptosis assay were performed in LX-2 cells and (B) primary human hepatic stellate cells (phHSC) exposed up to 100 nM AZD3355 for 72 hours showed no cytotoxic or apoptotic effect and normal cell proliferation. Results are reported as means ± SEM (n=3). *p<0.05, **p<0.01, ***p<0.001

**Figure S3. eXpression2Kinases Network.**

(A) Transcription factor enrichment analysis shows ranked list of the transcription factors predicted to regulate the list of differentially expressed genes. (B) Protein-protein interaction subnetwork connects the input list of enriched transcription factors (red) through intermediate proteins (gray). (C) Prediction of the protein kinases that are the likely regulators of the expanded protein-protein interaction network. (D) Inferred upstream regulatory network integrating the results of the transcription factor enrichment analysis, the protein network expansion and the kinase enrichment.

**Figure S4. Human liver slices tolerate up to 500 nM AZD3355 dosing.** Human precision cut liver slices (PCLS) were treated with either DMSO (as vehicle) or two different concentration of AZD3355 (250 and 500 nM) for 24 hours after which the culture media was harvested and secreted lactate dehydrogenase (LDH) was measured. No significant LDH leakage were found in AZD3355 treated liver slices compared to vehicle-treated group in all four patient samples .

**Figure S5. Effects of AZD3355 on body weight in NASH mice.** Both male and female mice treated with 30 mg/kg AZD3355 had significantly reduced body weight (A) beginning during second week (in male) or third week (in female) of treatment compared to the vehicle control group. Reduced body weight was not due to a toxic effect of the drug as no significant differences in western diet (B) or sugar water (C) intake were observed before and during administration of AZD3355 or vehicle (0.5% Methylcellulose). Vehicle: n = 9 male, 8 female; AZD3355 (10 mg/kg): n = 9 male, 8 female; AZD3355 (30 mg/kg): n = 9 male, 8 female; OCA (30 mg/kg): n = 9 male, 8 female animals. Results are reported as means ± SEM. *p<0.05, **p<0.01, ***p<0.001. OCA, obeticholic acid.

**Figure S6. Effects of AZD3355 on liver weights, necroinflammatory activity and lipid profile of NASH mice.** - Liver weight and Liver/body weight were measured at 24 weeks (after 12 weeks treatment). Significant reduction of liver weight and liver-to-body weight ratio by AZD3355 treatment compared to vehicle treatment in both male (A) and female (B) groups was observed. Dose dependent significant reduction of serum ALT, AST and triglycerides in male (C) or female (D) by AZD3355 treatment compared to vehicle-treated group indicated improvement of liver toxicity and necroinflammatory activity at 24 weeks (after 12 weeks of treatment). Vehicle: n = 9 male, 8 female; AZD3355 (10 mg/kg): n = 9 male, 8 female; AZD3355 (30 mg/kg): n = 9 male, 8 female; OCA (30 mg/kg): n = 9 male, 8 female. Results are reported as means ± SEM. *p<0.05, **p<0.01, ***p<0.001. ALT, alanine aminotransferase; AST aspartate aminotransferase; OCA, obeticholic acid

**Figure S7. Histological features and NAFLD activity scores of vehicle with drug treated NASH mice.** (A) Representative photomicrograph of H&E staining mouse liver used for assessment of NAFLD activity score (NAS). NAS-CRN (sum of steatosis, ballooning, lobular inflammation scores from Table 2) and fibrosis score were determined in male (B) and female mice (C). Significant reduction of fibrosis stage was observed by AZD3355 treatment compared to vehicle-treated animals in both male and female groups. OCA treatment significantly reduced both NAS and fibrosis stage. Vehicle: n = 9 male, 8 female; AZD3355 (10 mg/kg): n = 9 male, 8 female; AZD3355 (30 mg/kg): n = 9 male, 8 female; OCA (30 mg/kg): n = 9 male, 8 female animals. Black arrow = steatosis, yellow arrow = hepatocyte ballooning, red arrow = lobular inflammation. Results are reported as means ± SEM. *p<0.05, **p<0.01, ***p<0.001.

**Figure S8. Tumor development on AZD3355 treated NASH mice.** Photographs of male and female mouse livers showing surface appearances and tumor development (black arrow). In the vehicle-treated group (0.5% methylcellulose) the animals developed fibrosis and tumors. Both AZD3355 (10 mg/kg) and AZD3355 (30 mg/kg) or OCA (30 mg/kg) groups led to regression of fibrosis and reduced tumor numbers compared to the vehicle group

**Figure S9. Principle Components Analysis of RNAseq feature counts.** Feature counts from RNAseq samples were analyzed using Principle Components Analysis (PCA) after transformation with a variance-stabilizing transformation. The values of the first two principle components are summarized here for the 3 samples treated with AZD3355 and the 3 vehicle controls. Ex, Experiment

**Figure S10. Key fibrogenic protein expression of AZD3355 and OCA treated FAT-NASH mice.** Full length western blot (whole liver) images of either vehicle, AZD3355 or OCA treated mice represented in Figure 5

**Supplemental Figures:**

**Figure S1**


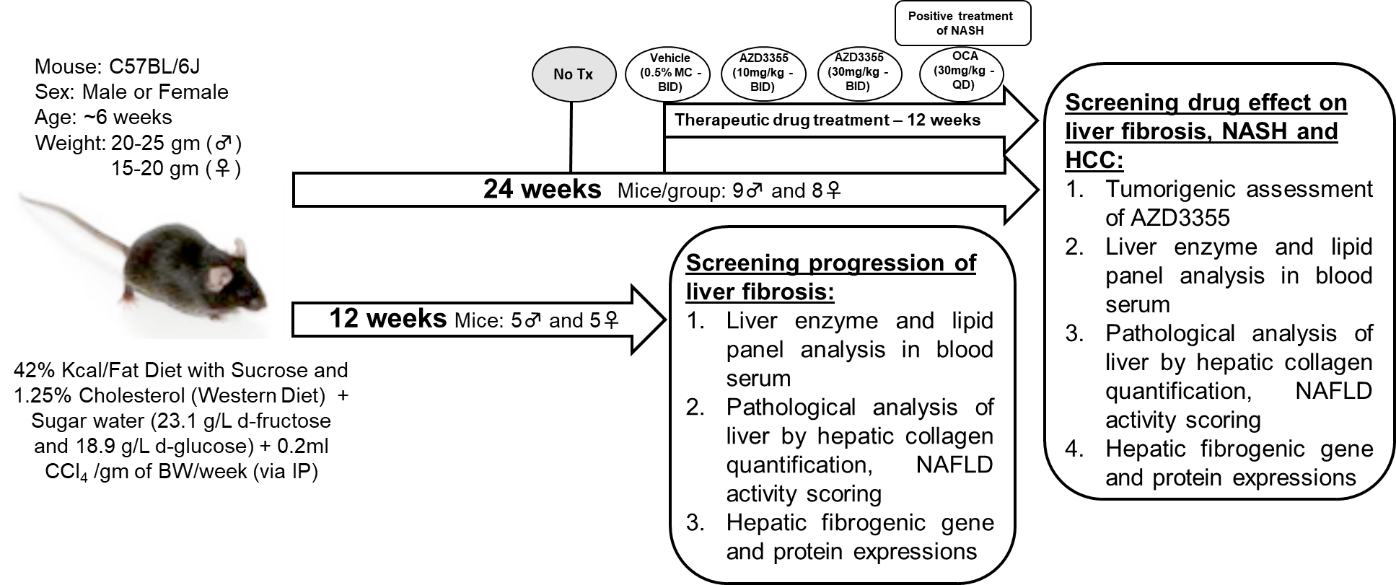


**Figure S2**

A


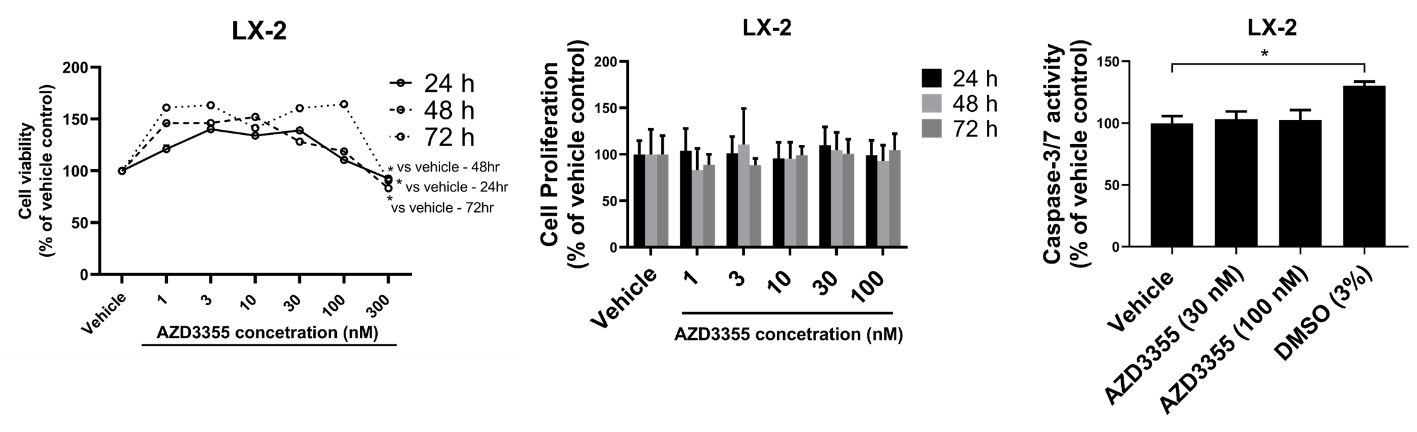


B


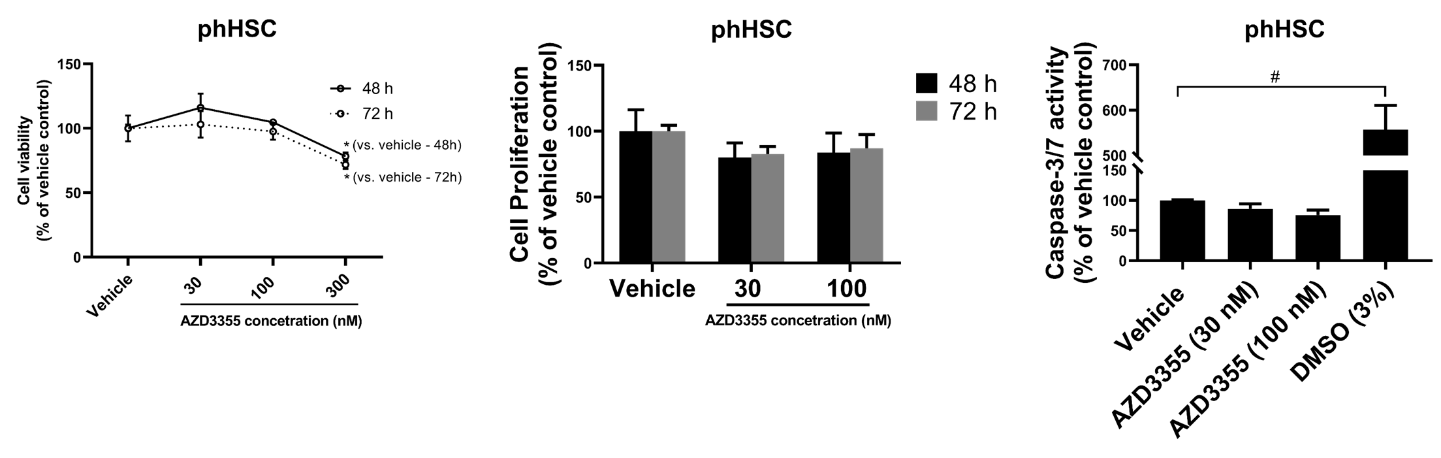


**Figure S3**

**
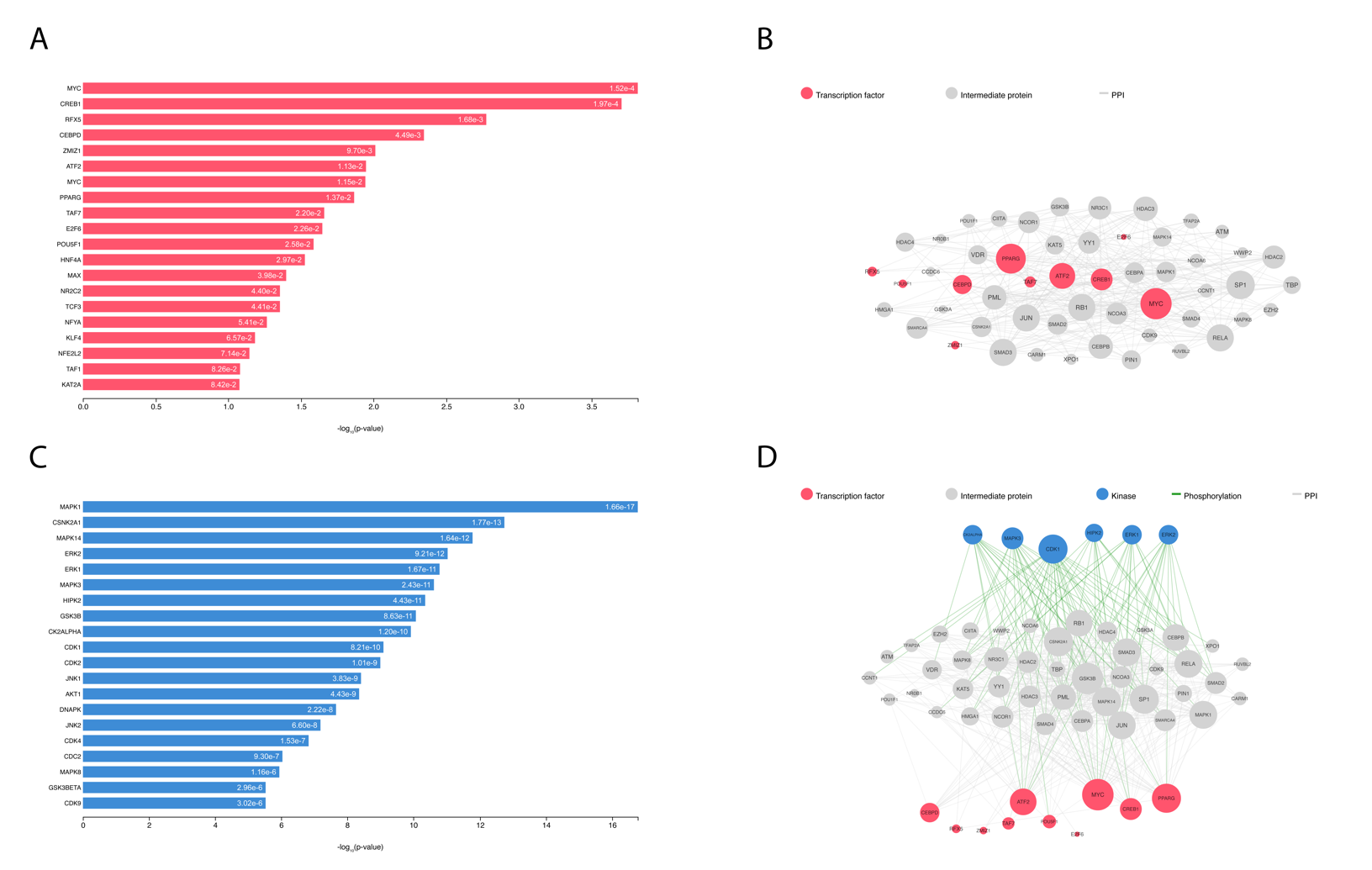
**

**Figure S4**

**Patient 1 Patient 2 Patient 3 Patient 4**

**
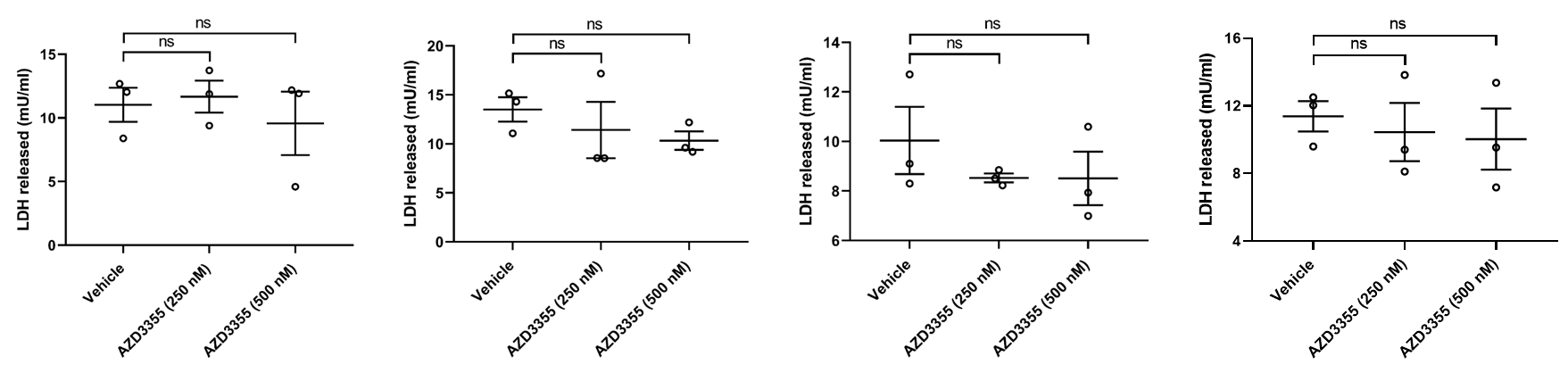
**

**Figure S5**

A


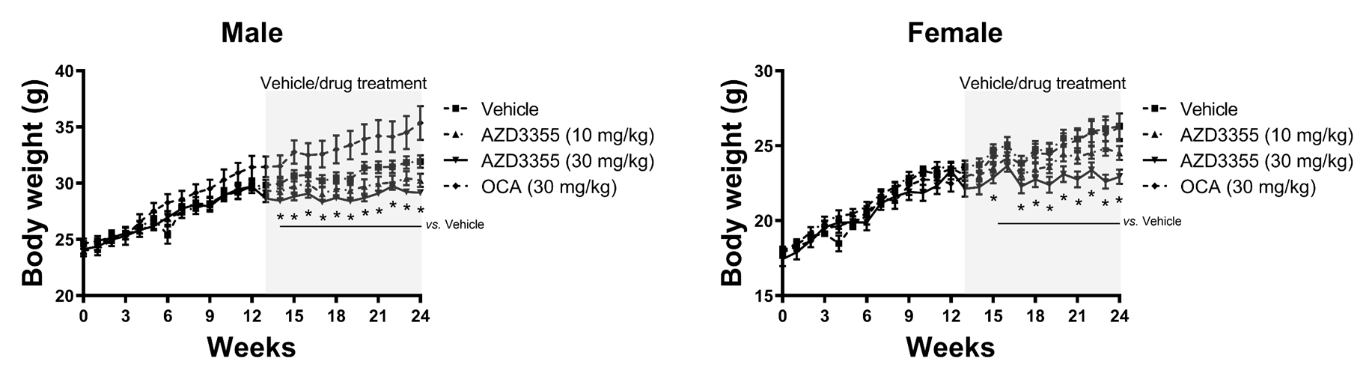


B


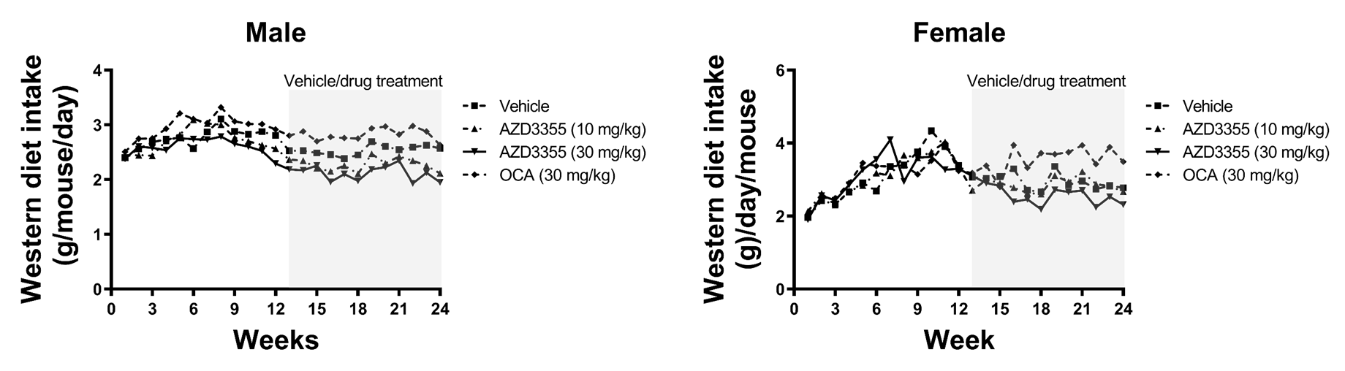


C


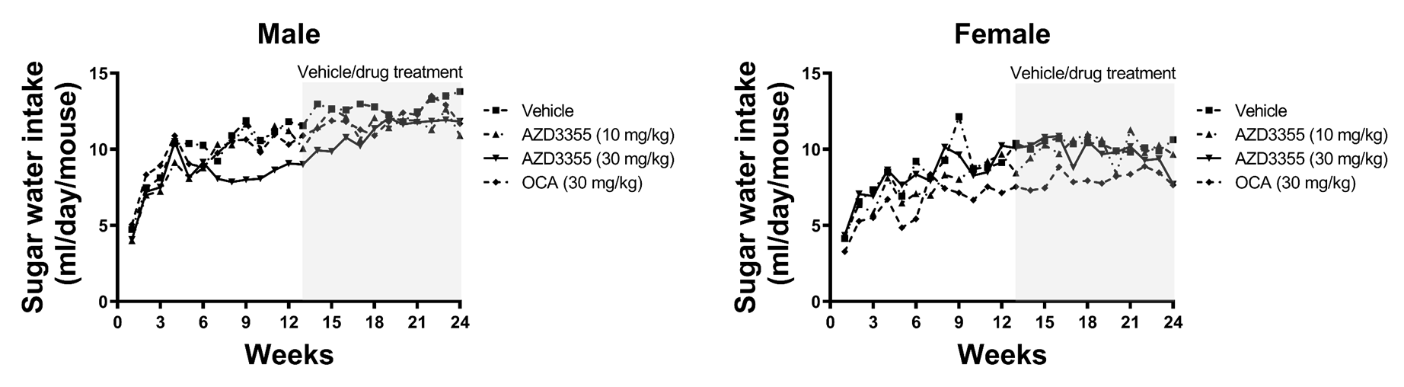


**Figure S6**

**A**


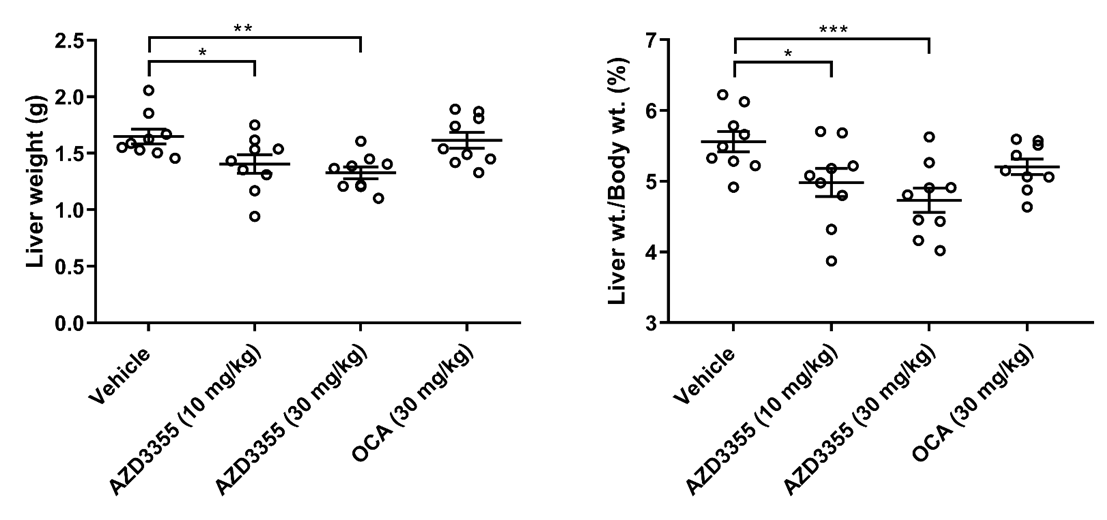


**B**

**
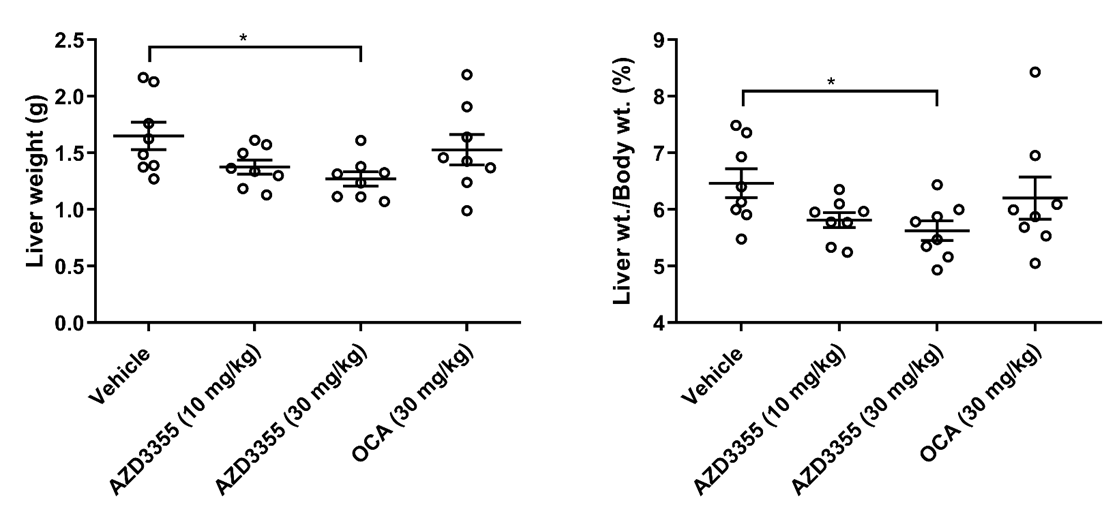
**

**C**


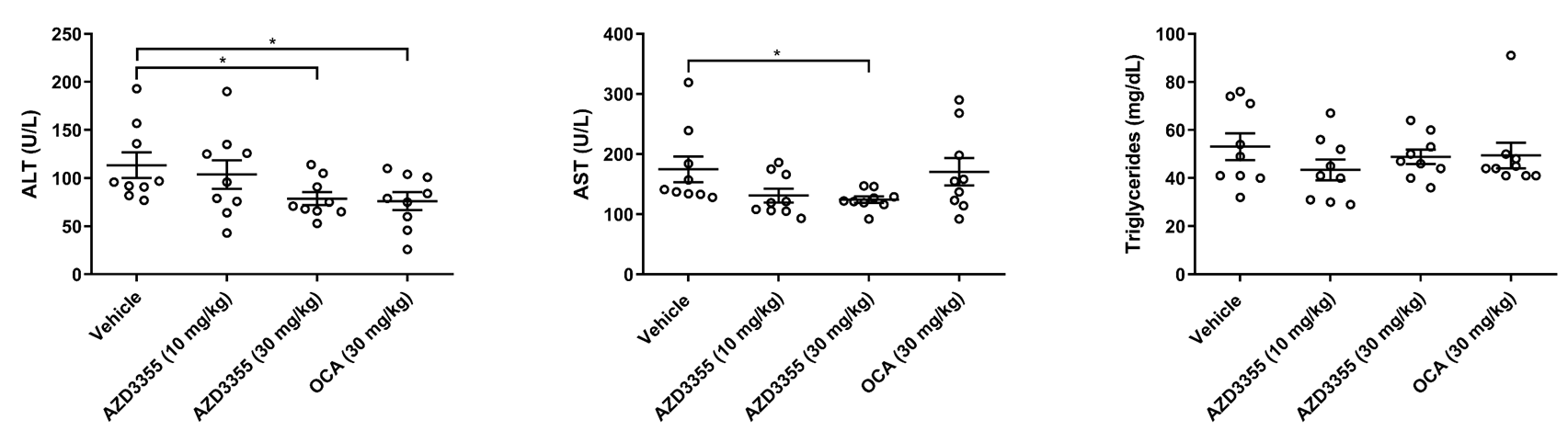


**D**


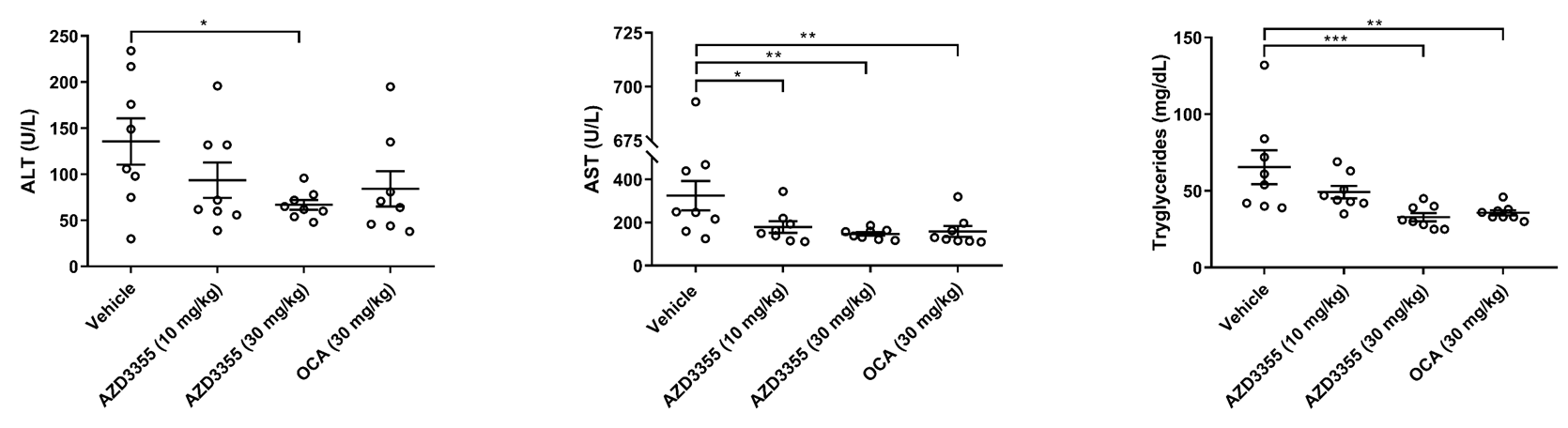


**Figure S7**

**
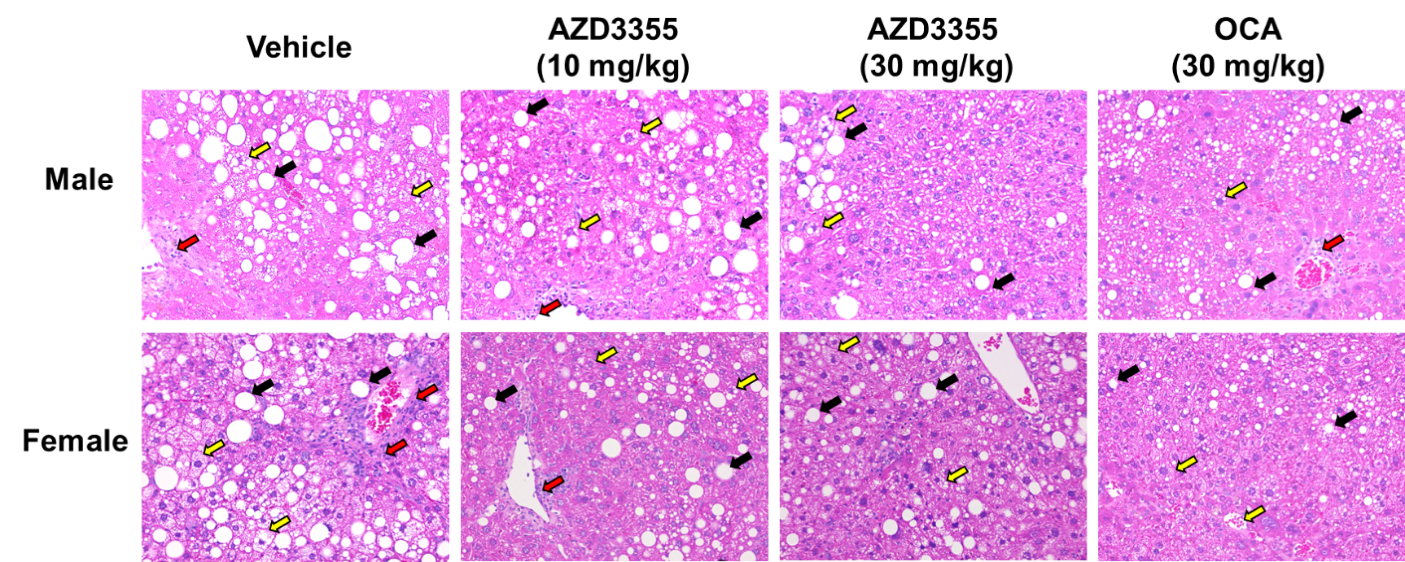
A**

**B.**


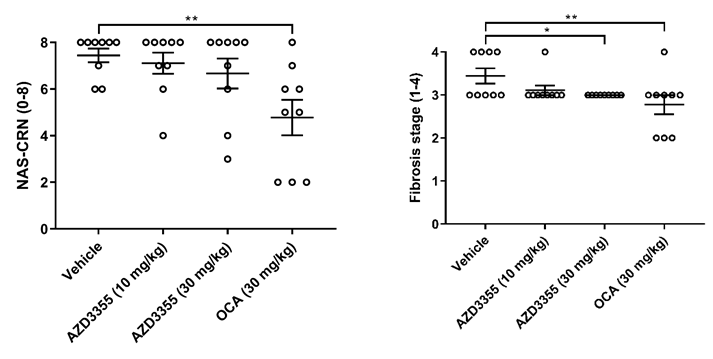


**C**

**
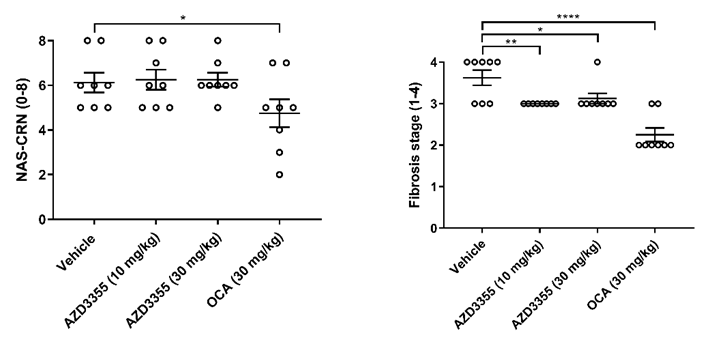
**

**Figure S8**

**
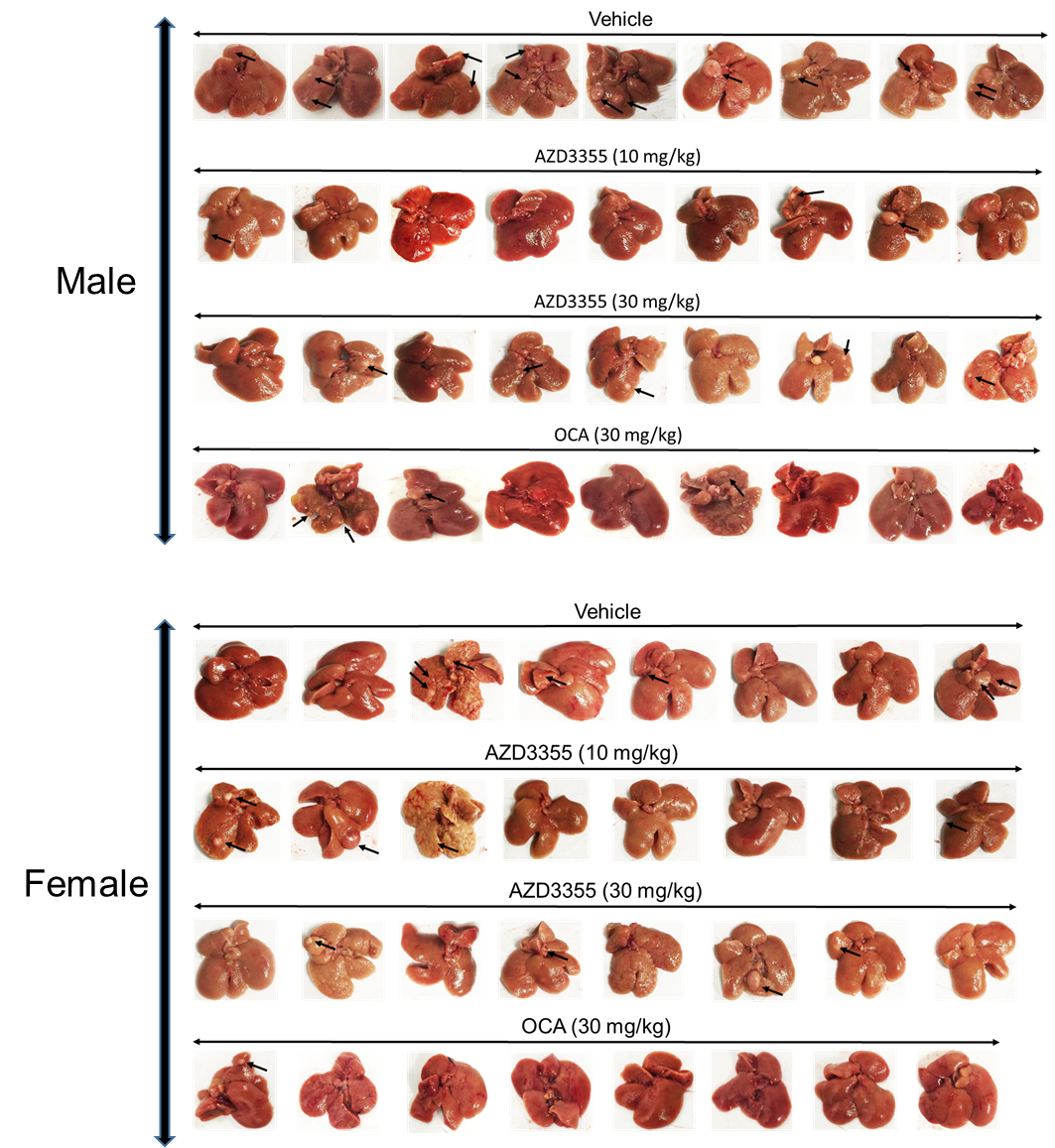
**

**Figure S9**

**
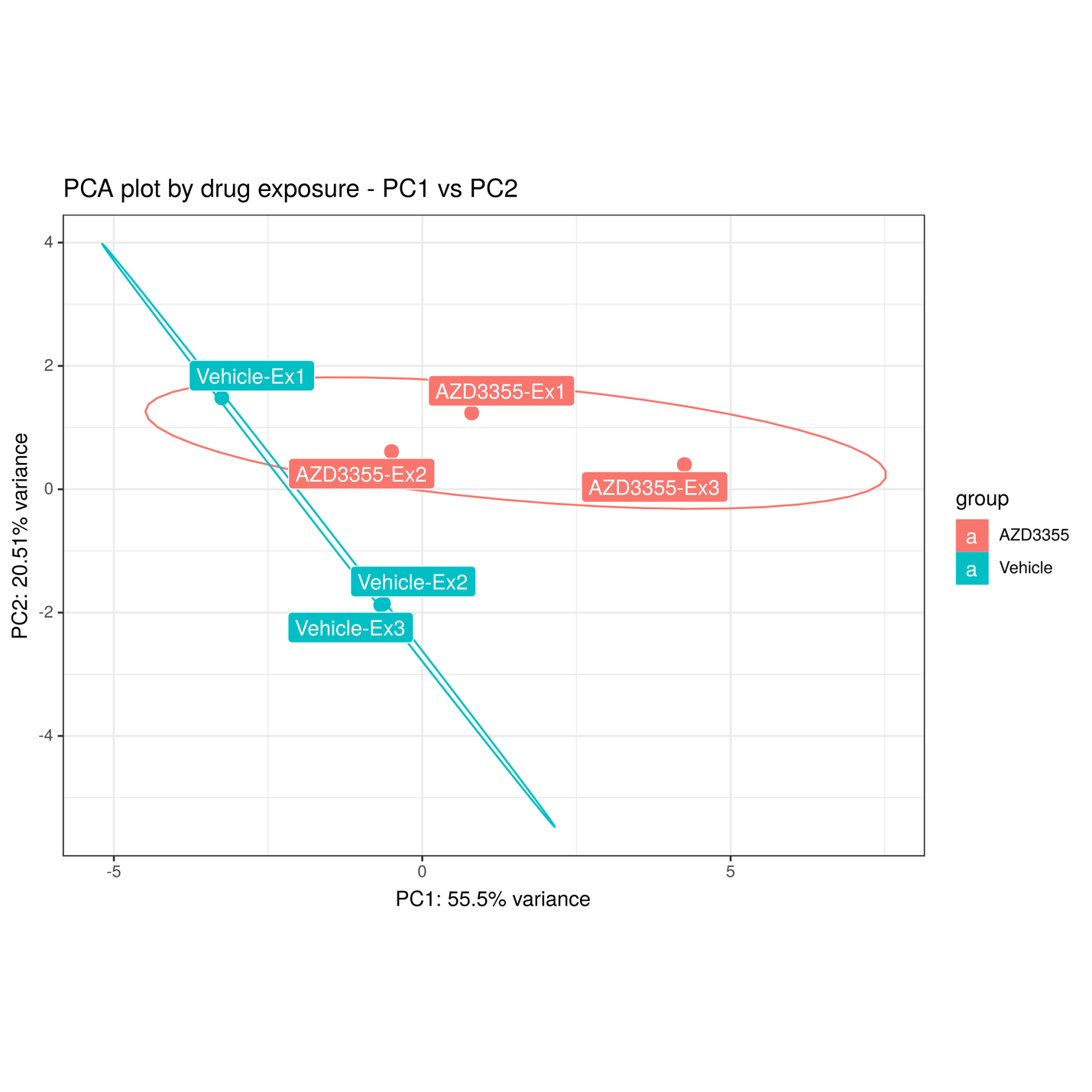
**

**Figure S10**

**
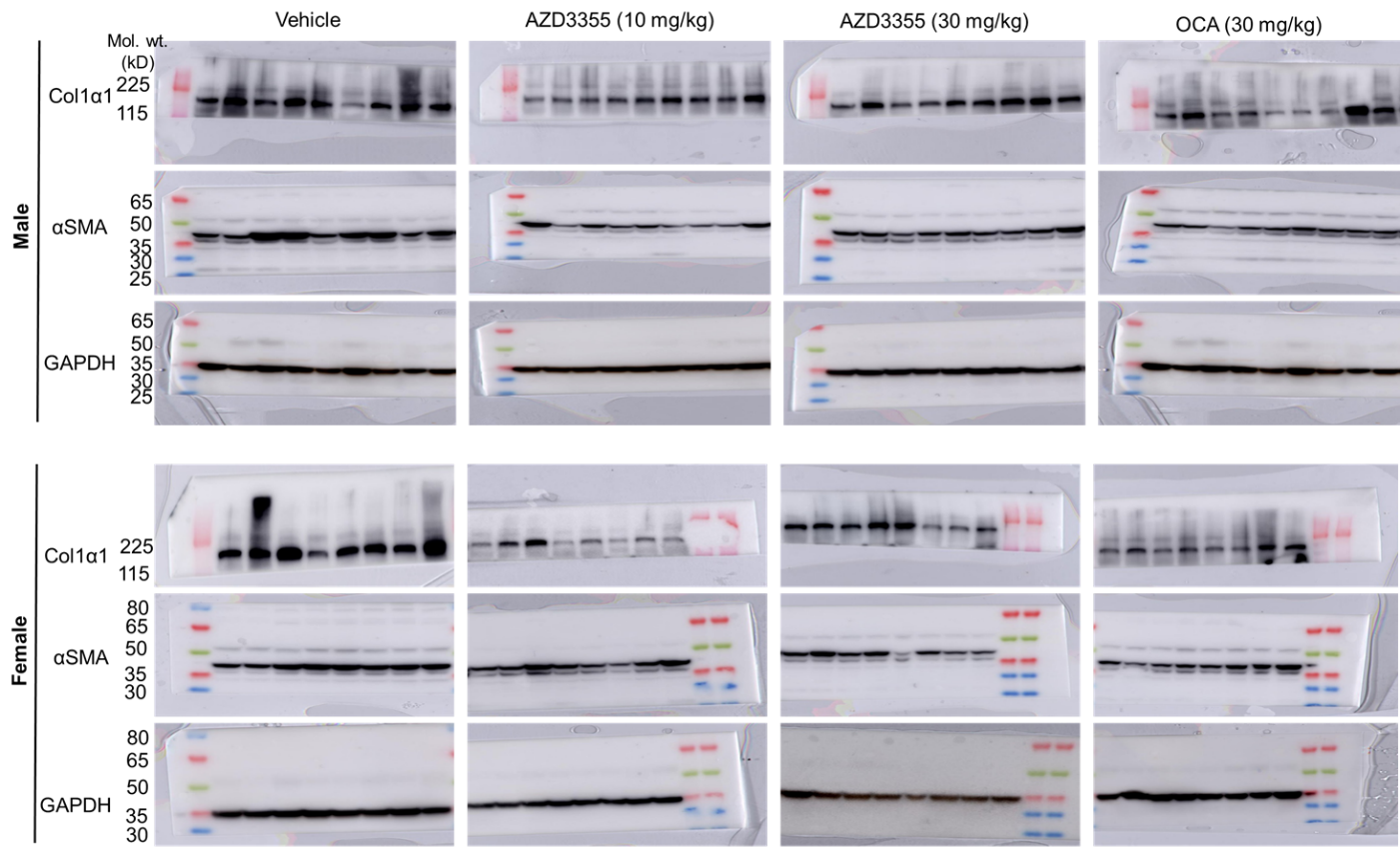
**
